# Population-level faecal metagenomic profiling as a tool to predict antimicrobial resistance in *Enterobacterales* isolates causing invasive infections: An exploratory study across Cambodia, Kenya, and the UK

**DOI:** 10.1016/j.eclinm.2021.100910

**Published:** 2021-05-30

**Authors:** Olga Tosas Auguet, Rene Niehus, Hyun Soon Gweon, James A. Berkley, Joseph Waichungo, Tsi Njim, Jonathan D. Edgeworth, Rahul Batra, Kevin Chau, Jeremy Swann, Sarah A. Walker, Tim E.A. Peto, Derrick W. Crook, Sarah Lamble, Paul Turner, Ben S. Cooper, Nicole Stoesser

**Affiliations:** a Centre for Tropical Medicine and Global Health, University of Oxford, Oxford, UK; b Harvard T.H. Chan School of Public Health, Harvard University, Boston, USA; c School of Biological Sciences, University of Reading, Reading, UK; d Centre for Ecology & Hydrology, Wallingford, UK; e KEMRI/Wellcome Trust Research Programme, Kilifi, Kenya; f The Childhood Acute Illness and Nutrition (CHAIN) Network, Nairobi, Kenya; g Centre for Clinical Infection and Diagnostics Research (CIDR), Department of Infectious Diseases, King's College London, London, UK; h Nuffield Department of Medicine, University of Oxford, Oxford, UK; i NIHR Health Protection Research Unit in Healthcare-associated Infections and Antimicrobial Resistance, Oxford, UK; j Wellcome Trust Centre for Human Genetics, University of Oxford, Oxford, UK; k Cambodia-Oxford Medical Research Unit, Microbiology Department, Angkor Hospital for Children, Siem Reap, Cambodia; l Mahidol–Oxford Tropical Medicine Research Unit, Faculty of Tropical Medicine, Mahidol University, Bangkok, Thailand

**Keywords:** Antimicrobial resistance surveillance, Metagenomics, Clinical infection

## Abstract

**Background:**

Antimicrobial resistance (AMR) in *Enterobacterales* is a global health threat. Capacity for individual-level surveillance remains limited in many countries, whilst population-level surveillance approaches could inform empiric antibiotic treatment guidelines.

**Methods:**

In this exploratory study, a novel approach to population-level prediction of AMR in *Enterobacterales* clinical isolates using metagenomic (Illumina) profiling of pooled DNA extracts from human faecal samples was developed and tested. Taxonomic and AMR gene profiles were used to derive taxonomy-adjusted population-level AMR metrics. Bayesian modelling, and model comparison based on cross-validation, were used to evaluate the capacity of each metric to predict the number of resistant *Enterobacterales* invasive infections at a population-level, using available bloodstream/cerebrospinal fluid infection data.

**Findings:**

Population metagenomes comprised samples from 177, 157, and 156 individuals in Kenya, the UK, and Cambodia, respectively, collected between September 2014 and April 2016. Clinical data from independent populations included 910, 3356 and 197 bacterial isolates from blood/cerebrospinal fluid infections in Kenya, the UK and Cambodia, respectively (samples collected between January 2010 and May 2017). *Enterobacterales* were common colonisers and pathogens, and faecal taxonomic/AMR gene distributions and proportions of antimicrobial-resistant *Enterobacterales* infections differed by setting. A model including terms reflecting the metagenomic abundance of the commonest clinical *Enterobacterales* species, and of AMR genes known to either increase the minimum inhibitory concentration (MIC) or confer clinically-relevant resistance, had a higher predictive performance in determining population-level resistance in clinical *Enterobacterales* isolates compared to models considering only AMR gene information, only taxonomic information, or an intercept-only baseline model (difference in expected log predictive density compared to best model, estimated using leave-one-out cross-validation: intercept-only model = -223 [95% credible interval (CI): -330,-116]; model considering only AMR gene information = -186 [95% CI: -281,-91]; model considering only taxonomic information = -151 [95% CI: -232,-69]).

**Interpretation:**

Whilst our findings are exploratory and require validation, intermittent metagenomics of pooled samples could represent an effective approach for AMR surveillance and to predict population-level AMR in clinical isolates, complementary to ongoing development of laboratory infrastructures processing individual samples.

**Research in Context**

**Evidence before this study**

Antimicrobial resistance (AMR) in *Enterobacterales* is a significant health threat, and individual-level surveillance remains difficult to implement systematically. Non-targeted, metagenomic approaches enabling prediction of population-level AMR from pooled minimally invasive clinical samples, could be of potential major public health benefit by: (i) enabling surveillance of AMR at the population-level; (ii) informing empiric treatment guidelines; and (iii) monitoring the impact of interventions. From a PubMed search using the terms “metagenomic”, “population”, “colonisation” and “infection” (01/Jan/2006–02/Dec/2020), 76 abstracts were evaluated and one full text reviewed; no studies identified used pooled colonisation samples from a population subset to infer phenotypic resistance in clinical isolates obtained from the same setting.

**Added value of this study**

This exploratory study described a metagenomics approach using pooled faecal/rectal samples from population subsets (~100–200 individuals) to predict setting-specific resistance in clinical *Enterobacterales* infections. We studied three geographic settings with different *Enterobacterales* AMR prevalence (Cambodia, Kenya, the UK) and three different age groups (neonates, children, adults). Taxonomy-adjusted AMR metrics combining taxonomic and AMR metagenomic profiles from pools, showed high out-of-sample prediction performance when considered in a Bayesian generalized linear model for their ability to predict the population-level prevalence of AMR in clinical isolates, compared to other metrics or no metrics.

**Implications of all the available evidence**

Whilst further validation of this exploratory novel approach is needed, it shows potential to rapidly overcome a lack of quality AMR surveillance data and inform empiric treatment guidelines, particularly in LMICs where surveillance infrastructures remain sparse. It could be evaluated for other priority bacteria and different colonisation samples. Surveillance based on population colonisation metagenomics and taxonomy-adjusted AMR metrics should be further evaluated for its potential public health benefit in combatting AMR and supporting antibiotic stewardship.

Alt-text: Unlabelled box

## 1 Introduction

Antimicrobial resistance (AMR) is a global health emergency [1], particularly in resource-limited settings, where effective microbiological services and antibiotics may be unavailable [2]. Surveillance is key to mitigating the effects of AMR by monitoring trends, informing empiric treatment guidelines, identifying emerging threats, and evaluating the impact of interventions. There has been significant investment in individual/patient-level surveillance, and an attempt to promote standardised collection, analysis and sharing of global AMR data, capturing both clinical and microbiological information [3]. However, limitations of these approaches include developing and sustaining robust capacity in regions where AMR is most prevalent, and in obtaining systematic data even from countries with adequate infrastructure. Population-level surveillance strategies complementary to the implementation of such individual-level approaches would be of benefit.

Colonisation with specific species and/or drug-resistant organisms, such as nasal colonisation with *Staphylococcus aureus* [4], or rectal colonisation with carbapenemase-producing *Enterobacterales*, [5] is associated with infection risk. Metagenomic approaches are less biased than culture-based approaches, and metagenomic AMR gene abundances and taxonomic distributions have been used as correlates for national antibiotic exposures and AMR monitoring in sewage [[6], [7], [8], [9], [10]]. However, to our knowledge, no study has used taxonomic and AMR gene profiles in pooled metagenomes to directly estimate AMR prevalence amongst clinical isolates in populations in the same setting. This approach would enable intermittent, strategic sampling of a population subset to estimate the burden of AMR in clinical isolates. Most colonisation sites are easy-to-sample and sampling is well-tolerated (e.g. faeces/rectal swabs).

The concept of a metagenomic taxonomy-adjusted AMR metric or “resistance potential” has been described previously [6,11] as the average metagenome fraction encoding AMR genes for a particular antibiotic or class, across all bacterial taxa in a sample that can potentially carry such AMR genes, based on assumed taxonomic ranges for respective AMR gene families. As a proof-of-principle study we sequenced pooled faecal samples from a population subset of >100 individuals in three disparate geographic settings with varying *Enterobacterales* AMR prevalence and infection rates, namely Cambodia, Kenya and the United Kingdom (UK), creating three pooled population metagenomes. We developed a set of Bayesian generalised linear models - each using different combinations of taxonomy and AMR metagenomic metrics derived from these metagenomes - to predict AMR prevalence at a population-level in clinical *Enterobacterales* isolates in each setting. Bayesian model comparison was then used to determine the value of metagenomic taxonomy-adjusted AMR metrics for out-of-sample predictive accuracy in determining the prevalence of AMR in clinical *Enterobacterales* isolates .

## 2 Methods

### 2.1 Samples and settings

Faecal material collected after 2014 at pre-admission clinics or on admission to hospital from three age-groups and settings was studied, namely: children 1–59 months in Kilifi, Kenya; newborns in Siem Reap, Cambodia; and adults ≥18 years in London, UK (appendix p2). Rectal swabs and faecal samples have both been used as approaches for intestinal metagenomics [12,13], and give comparable results [14].

For each study site, microbiology metadata for blood/cerebrospinal fluid samples (as most robustly representative of truly invasive infections) collected within 0–72 h of admission to hospital (i.e. community-associated) from 01/Jan/2010–31/May/2017 were collated. Each site has a microbiology laboratory participating in external quality assurance schemes and accreditation processes (appendix p2). Samples were processed using standard operating procedures in accordance with international guidelines (appendix p2). Collated metadata included bacterial species and antibiotic susceptibility test (AST) results, specimen type, and basic patient details to validate aggregate-level stratification by age. Infection metadata were collated for individuals < 90 days of age in Cambodia, ≤ 60 months of age in Kenya and ≥ 18 years of age in the UK (appendix pp2-3).

### 2.2 DNA extraction

DNA was extracted from each sample using the MoBio PowerSoil® DNA isolation kit (Qiagen, Hilden, Germany), as per the manufacturer's instructions with optimisation steps to achieve sufficient DNA yields for sequencing (ideally ≥300 ng DNA/34ul, with a view to obtaining ≥20Gbp (Giga base pairs) of data per sample; appendix pp4-8).

### 2.3 Sample pooling

DNA extracts were stored at −20 °C prior to pooling and sequencing. For each study setting, we created a “population pool”, which consisted of the pooling of equimolar concentrations of all extracts from that setting with ≥1 ng DNA/μl. To validate our pooling approach (see appendix pp11, 18-19), we also created one smaller pool for each setting, a “30-sample pool”, which consisted of equimolar concentrations of 30 randomly selected extracts with ≥300 ng DNA/34μl. An aliquot from each extract included in the 30-sample pools was also sequenced individually for the validation (i.e. sequenced extracts from 90 individuals in total). An aliquot from all extracts sequenced individually and included in the 30-sample pools was also included in the population pools.

### 2.4 Metagenomic sequencing

Sequencing of all faecal sample DNA extracts (pools and individual extracts) was performed using the HiSeq 4000 Illumina platform, generating 150 bp paired-end reads (i.e. 96 metagenomes [*n* = 90 individual metagenomes, *n* = 3 30-sample pools, *n* = 3 population pools]; appendix p9).

### 2.5 Sequence data processing

Taxonomic abundance of bacterial species and AMR genes at individual and pooled sample levels was determined using a published bioinformatics pipeline, ResPipe [15]. This pipeline incorporates established approaches to taxonomic profiling, namely Kraken2 [16] and Bracken [17], and an adapted approach to quantifying AMR gene markers present in a metagenome by mapping sequences against the Comprehensive Antibiotic Resistance Database [18,19] (CARD, v.3.0.3) (for method details, see appendix p9). All AMR genes identified in any of the samples were included in the analysis. Since ResPipe reports the number of sequences that mapped to each AMR, in order to remove reference gene length bias, the AMR gene profiles - i.e. the numbers of sequences mapping to each AMR gene - were corrected using the following formula: corrected gene count (CGC) = (specific read count x average read length) / (AMR gene length x specific lateral coverage) where (1) *specific read count* is the number of sequences mapping exclusively to the reference AMR gene; (2) *specific lateral coverage* is the proportion of the AMR gene covered by sequences mapping exclusively to the gene; (3) *AMR gene length* is the length of the gene the sequence is mapped to; and (4) *average read length* is average length of reads that mapped to the AMR gene.

The CARD database classifies each reference AMR gene by its association with phenotypic resistance. To be in CARD, an AMR gene must be described in a peer-reviewed scientific publication, have its DNA sequence available in GenBank, and include experimental evidence of elevated minimum inhibitory concentration (MIC) over controls. [19] We used these data to map and aggregate counts of AMR genes/variants associated with resistance to specific antibiotics. We ranked the AMR genes/variants into two categories, reflecting to some extent the public health risks posed [20]. The first category, AMR_DEF_, included only AMR determinants with the CARD “*Confers_Resistance_to_Antibiotic*” relationship ontology term, whereby the gene is known to confer or contribute to clinically relevant resistance to a specific antibiotic [19]. The second category, AMR_ALL_, contained corrected counts of all AMR determinants with clear experimental evidence of increasing the MIC, including those associated with clinically relevant resistance (as for AMR_DEF_), but also those without the definitive “*Confers_Resistance_to_Antibiotic*” relationship ontology term. In this study we have used “antimicrobial resistance (AMR) gene” to define any relevant marker of resistance, including genes that confer resistance by mutation (but can have a susceptible wild type), and genes that confer resistance through presence/absence.

### 2.6 Taxonomy-Adjusted antimicrobial resistance (AMR) metrics

We developed AMR metrics and taxonomy metrics from pooled metagenomes (i.e. “population pools”) to predict the number of resistant isolates causing infection in each setting. Resistance metrics (*R_CGC_*) aim to summarise information about relative abundance of genes conferring resistance for a given antibiotic. These are given through the sum of corrected gene counts (CGCs) of AMR gene variants associated with resistance to a given antibiotic, *j (R_CGCj_*), divided by the total CGC of all AMR genes in the pool. We considered two possibilities. First, *R_CGC_DEF_* considered only AMR determinants known to definitively confer clinically relevant resistance (using AMR_DEF_, as defined above). Second, *R_CGC_ALL_* was calculated for AMR determinants with clear experimental evidence of increasing the MIC (using AMR_ALL_). Taxonomic metrics (*R_Tax_*) aim to represent relative taxonomic abundance, and were given through the estimated abundance of a clinically relevant bacterial grouping divided by the total estimated abundance of bacterial taxa in the pool. Three bacterial groupings were evaluated: (i) the entire *Enterobacterales* order (*R_Tax_E_*); (ii) species in the *Enterobacteriaceae* family only (*R_Tax_e_*); and (iii) the grouping of the four most common and clinically relevant bacterial genera/species within the *Enterobacteriaceae* family across study sites (namely *Escherichia coli, Klebsiella pneumoniae, Salmonella* spp., *Enterobacter* spp.; (*R_Tax_e4_*)). Our prediction models included at most one taxonomic and one resistance metric, and evaluated six resistance and taxonomy metric combinations in total. We refer to these six combinations as taxonomy-adjusted AMR metrics.

### 2.7 Statistical analysis

For each of the six taxonomy-adjusted AMR metrics, we fitted a Bayesian generalized linear model to the infection data and applied Bayesian model comparison based on out-of-sample prediction accuracy. This allowed us to assess the potential of each metric to predict antibiotic resistance amongst clinical invasive *Enterobacterales* isolates and to determine the most predictive metric. Using cross-validation as a tool for model comparison penalizes models that are overfitting the data. We used de-duplicated counts of isolates (unique bacterial species per antibiogram and patient-ID) for the analyses. We let *i* denote the setting (Cambodia, Kenya or UK), and *j* the antibiotic being evaluated. We assumed that counts of resistant isolates follow a binomial distribution. Our model then predicts the count of resistance (*r_i,j_*) amongst tested *Enterobacterales* isolates (*n_i,j_*) using a probability of resistance (*p_i,j_*; see appendix pp9-11 for details of model equations and parameters).

(1) $$\text{logit}\left(p_{i,j}\right)=\alpha_{j}+\beta_{1,j}R_{\text{CGC},i,j}+\beta_{2,j}R_{\text{Tax},i}$$

We included only those antibiotics that had existing antibiotic susceptibility test (AST) data in ≥2 of three settings; missing observations were excluded from the likelihood evaluation. Due to the limited number of infection isolates with AST results (especially in Cambodia), we chose standard weakly informative priors for the intercept (α_j_) and the effect parameters (*β*_1_*_,_*_j_, *β*_2_*_,_*_j_; appendix pp9-11). In addition, we restricted the effect of AMR gene abundance to be positive, reflecting our view that only a positive association of AMR genes and clinical resistance is biologically reasonable. Each model was fit using Stan software [21] (v2.19.1), taking 50,000 samples after a burn-in period of 5000 samples using four independent chains. We assessed chain convergence by inspecting chain traceplots and ensuring small values of the R-hat chain convergence diagnostic (R-hat<0.01) [22].

The best taxonomy-adjusted AMR metric was selected using Bayesian leave-one-out cross validation [23], which estimates the model's pointwise out of sample prediction accuracy. Importantly, as opposed to assessments of within sample prediction, leave-one out cross-validation estimates how well a model is expected to predict new, unseen data points, and it thus penalizes models that are overfitting. In addition to the six model versions with the different taxonomy-adjusted AMR metrics, we also included in the comparison one baseline model with resistance metrics (*R_CGC_*) but without taxonomy metrics (*R_Tax_*), one with only taxonomy metrics (*R_Tax_*), and a baseline (null) model with only intercepts (keeping only αj). This was to assess the value of considering only taxonomy or only AMR metrics (i.e. whichever *R_Tax_* or *R_CGC_* metric performed best in the models considering combinations), against the value of combining these into a taxonomy-adjusted AMR metric for predicting clinical resistance. The within sample fit of the best model (chosen based on cross-validation) and of the null model, was assessed using logarithmic scoring of the posterior predictions against the observed counts of resistant isolates. This type of scoring assigns low scores to models with highly diffuse (uncertain) predictive distributions, and also to narrow but wrongly placed distributions, and is rooted in information theory and the definition of entropy [24]. For settings and antibiotics where zero samples were tested, we imputed the sample size by computing the rounded mean of the sample sizes of the other two settings. Model comparisons and all further data analyses were performed in R-3.6.1 statistical software [25].

### 2.8 Ethical approvals

Ethical approval for faecal/rectal samples was already in place (KEMRI/SERU/CGMR- C/023/3161, OXTREC 47-15 [26]; OxTREC ref 1047-13 [27]; and REC: 14/LO/2085 [28,29]). Samples were only collected from patients who provided informed consent or, in the case of children, whose parents/guardians provided consent on their behalf. This study was approved by the Oxford Tropical Research Ethics Committee (Reference: 5126-16), with additional local ethics clearance for inclusion of Cambodian and Kenyan samples, and a substantial amendment to 14/LO/2085 (NRES London-Camberwell St Giles) for UK samples.

### 2.9 Role of funding source

The funders and sponsor of the study had no role in study design, data collection, data analysis, data interpretation, or writing of the report; the views expressed are those of the authors and not necessarily those of the funders or the sponsor. All authors had full access to all the data in the study and had final responsibility for the decision to submit for publication.

## 3 Results

### 3.1 Metagenomic sequencing outputs

**Fig. 1:**
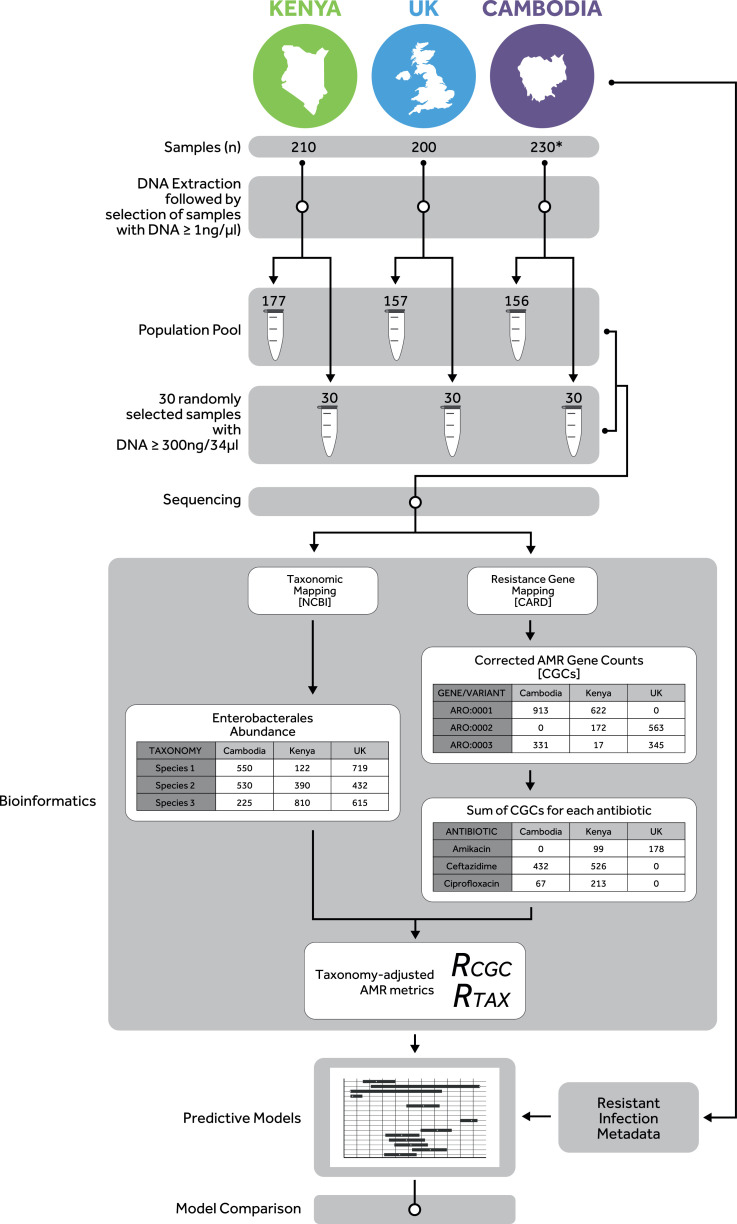
*Overview of sample and data collection and study methods.* The study collated human faecal material from existing biobanks in Kenya, UK and Cambodia. Collections comprised 210, 200 and 230 samples from Kenya (Apr-Sep 2016), UK (Feb-May 2015) and Cambodia (Sep 2013-Sep 2014), respectively. Following DNA extraction, samples with ≥1 ng/μl were used to create a metagenomics population pool from each setting. Amongst these, 30 samples with ≥ 300 ng/34μl were randomly selected to also be individually sequenced and to create a 30-sample pool, for a pooling validation study (see appendix pp11, 18-19). Each setting provided microbiology and AST results from hospital laboratory information systems (LIS), for blood and cerebrospinal fluid clinical samples collected on admission to the same hospitals over a seven-year period (2010–2017). DNA samples were sequenced using HiSeq 4000 Illumina platform; 150 bp paired-end reads were quality-filtered using a recently developed bioinformatics pipeline [15]. Sequences were mapped against NCBI for profiling the abundance of bacterial species, and against the Comprehensive Antibiotic Resistance Database (CARD) [18,19] for profiling antimicrobial resistance (AMR) genes/variants. The number of sequences that mapped to each AMR gene were corrected to remove resistance gene length bias, by computing corrected gene counts (CGCs). The CGCs were then aggregated according to the antibiotic these conferred resistance to. Several combinations of resistance (*R_CGC_*) and taxonomy (*R_Tax_*) abundance metrics were considered in a Bayesian modelling analysis, to assess the potential of each metric to predict antibiotic resistance amongst clinical invasive *Enterobacterales* isolates observed from LIS data in the three settings.

To enable efficient indexing, this study considered only samples with DNA yields ≥1 ng/ul (79–89% of hospital admission samples). Population pools in Kenya, the UK and Cambodia therefore comprised 177, 157, and 156 pooled faecal sample extracts (Fig. 1). The total Gbp of data per population pool was 51.6 (Kenya), 55.1 (UK) and 52.6 (Cambodia). The median Gbp for individually sequenced samples was 24.2 (Kenya), 22.1 (Cambodia) and 22.4 (UK). The following sections report the results of the analysis of population pools. The results of the validation of our pooling approach, which also consider metagenomic information from 30-sample pools and individual samples to assess whether pooled metagenomes are a fair representation of the individual metagenomes, are provided in the supplementary appendix (pp 18-19).

### 3.2 Taxonomic profiling of population metagenomes

**Fig. 2:**
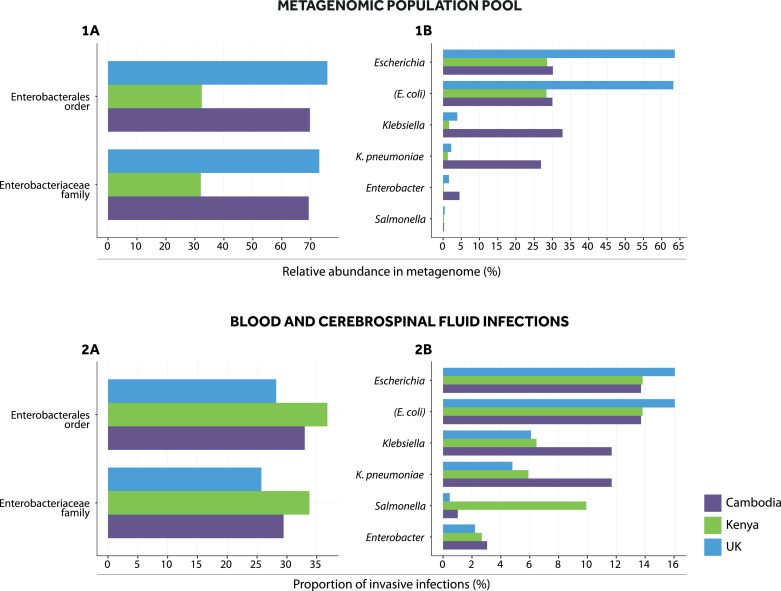
*Major Enterobacterales in metagenomic population pools and in bloodstream and cerebrospinal fluid infections.* The figure shows relative abundances of *Enterobacterales* in metagenomic population pools and proportions of blood and cerebrospinal fluid infections caused by major *Enterobacterales* in Cambodian, Kenyan and UK study settings. Panels for metagenomic population pools (*1A, 1B*) show, for each setting, the abundances of *Enterobacterales* taxa divided by the total abundance of bacterial taxa in a pool. Abundances are derived from Bracken estimates. Panels for invasive infection data (*2A, 2B*) show the proportion of bloodstream and cerebrospinal fluid isolates that were *Enterobacterales* out of all bloodstream and cerebrospinal fluid isolates with speciation results in target age groups, in each setting, from 2010 to 2017 (Cambodia [*n* = 197]; Kenya [*n* = 910]; UK [*n* = 3356]).

*Enterobacterales* were the most abundant bacterial taxa identified in the UK (75.7%) and Cambodia (69.7%) but not Kenyan (32.4%; Fig. 2, panel *1A*) population metagenomes, consistent with previous Kenya data [30]. Amongst *Enterobacterales*, >95% abundance was attributable to the *Enterobacteriaceae* family, across settings (UK: 96.3%; Cambodia: 99.4%; Kenya: 99.1%) (Fig. 2, panel *1A*). The most abundant *Enterobacterales* genera/species were *E. coli* and *K. pneumoniae*, followed by *Enterobacter* spp. (Fig. 2, panel *1B*; 92.4% of all *Enterobacterales* taxa in Kenya, 88.5% in the UK, 88.1% in Cambodia). The abundance of *E. coli* was >20-fold higher than that of *K. pneumoniae* in UK (*E. coli*: 63.2%; *K. pneumoniae*: 2.2%) and Kenyan (*E. coli*: 28.4%; *K. pneumoniae*: 1.3%) metagenomes, reflecting the fact that in general, *E. coli* is thought to be a more dominant gastrointestinal coloniser than *K. pneumoniae*. In contrast both species had similar abundance in the Cambodian metagenome (*E. coli*: 30%; *K. pneumoniae*: 26.9%), consistent with previous high rates of *K. pneumoniae* colonisation identified on culture in the neonatal group studied [27]. *Enterobacter* spp. abundance was also higher in Cambodia (4.5%) compared to the UK (1.6%) or Kenya (0.2%). Other *Enterobacterales* genera represented <2% of bacterial abundance across settings (appendix p12).

### 3.3 Enterobacterales isolates causing bloodstream and cerebrospinal infections

Amongst 197, 910 and 3356 bacterial isolates cultured from blood/cerebrospinal fluid infections in Cambodia, Kenya and the UK, respectively, infections by *Enterobacterales* accounted for approximately a third across settings (Kenya: 36.8%; Cambodia: 33.0%; UK: 28.2%) (Fig. 2, panel *2A*). Similar to the population-pool metagenomic data, most of these involved *Enterobacteriaceae* (UK: 91.2%, Cambodia: 89.2%; Kenya: 91.8%; Fig. 2, panel *2A*), and specifically *E. coli* and *K. pneumoniae*, with higher proportions of *E. coli* infections in the UK (*E. coli*: 16.1%; *K. pneumoniae*: 4.8%) and Kenya (*E. coli*: 13.8%; *K. pneumoniae*: 5.9%), versus Cambodia (*E. coli*: 13.7%; *K. pneumoniae*: 11.7%) (Fig. 2, panel *2B*). *Enterobacter* spp. was the next most common *Enterobacterales* genus causing infection across settings (Cambodia: 3.1%; Kenya: 2.7%; UK: 2.2%), with other *Enterobacterales* genera accounting for <2% of the total invasive infections (appendix p13). A notable exception was *Salmonella* spp., accounting for 9.9% of the total infections in Kenya (therefore included in Fig. 2, panels *1B, 2B*), consistent with data supporting the high rates of non-typhoidal salmonellosis here [31].

### 3.4 Metagenomic AMR gene profiling

We identified 863 different AMR genes across all samples/pools, including those known to confer clinically-relevant resistance for 113 antimicrobials (AMR_DEF_) and to increase the MIC for 163 antimicrobials (AMR_ALL_). A specific evaluation of AMR gene richness, on rarefied data (appendix p9), demonstrated that the number of AMR genes in population pools and individual samples differed by geographical setting, being highest in Cambodia (appendix p18).

**Fig. 3:**
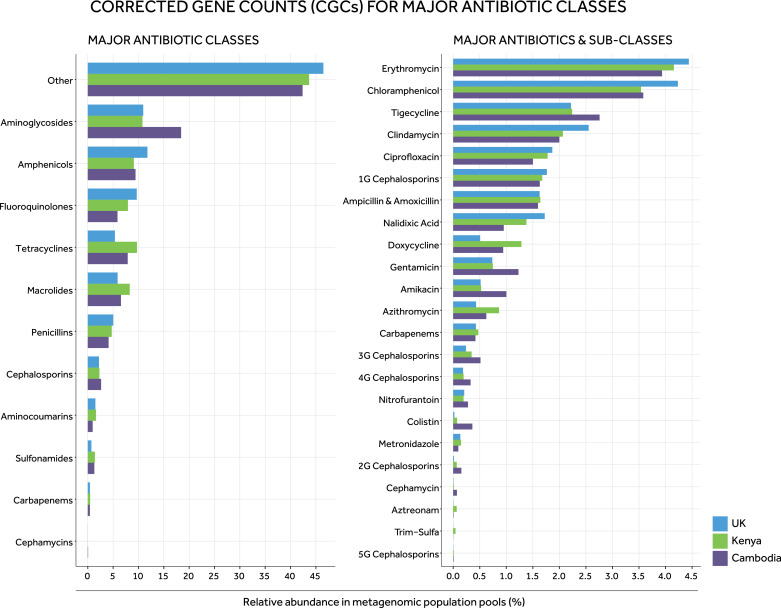
*Relative abundance of AMR genes (corrected gene counts [CGCs]) in metagenomic population pools.* Panels show, for each setting, corrected resistance gene counts (CGCs) for major antibiotic classes (left-hand panel), or antibiotic sub-classes/types (right-hand panel), divided by the total corrected AMR gene counts identified in the population pool. Relative abundances were calculated using *R_CGC_ALL_*, which considers corrected counts of genes and variants (CGC) increasing the MIC or conferring clinically relevant resistance for a given antibiotic. “Trim-sulfa” is trimethoprim-sulfamethoxazole; <number>*G* denotes the generation of cephalosporin (e.g. 1G represents first generation cephalosporins).

In the population metagenomes, the highest relative AMR gene abundances were for those associated with resistance to aminoglycosides, amphenicols, fluoroquinolones, tetracyclines and macrolides (48.1%, 45.8% and 43.6% of total AMR gene counts in Cambodia, Kenya and the UK, respectively) (Fig. 3, left-hand panel). However, the relative abundance of these differed between settings. For example, the relative abundance of AMR genes for aminoglycosides in Cambodia (18.4%) was almost double that in Kenya (10.8%) or the UK (10.9%). The next highest relative abundance was of genes conferring resistance to penicillins (Cambodia: 4.1%; Kenya: 4.7%; UK: 5.0%) and cephalosporins (Cambodia: 2.6%; Kenya: 2.3%; UK: 2.2%). AMR gene counts for other antibiotic classes were <2% of the total gene counts across settings, including to carbapenems (Kenya [0.5%], Cambodia and the UK [0.4%]). For single antibiotics or antibiotic sub-classes (e.g. 1st generation cephalosporins), the highest relative abundances were observed for erythromycin (Cambodia: 3.9%; Kenya: 4.2%; UK: 4.4%) and chloramphenicol (Cambodia: 3.6%; Kenya: 3.5%; UK: 4.2%) in all settings (Fig. 3, right-hand panel).

### 3.5 Susceptibility phenotypes of *Enterobacterales* isolates causing bloodstream and cerebrospinal infections

**Fig. 4:**
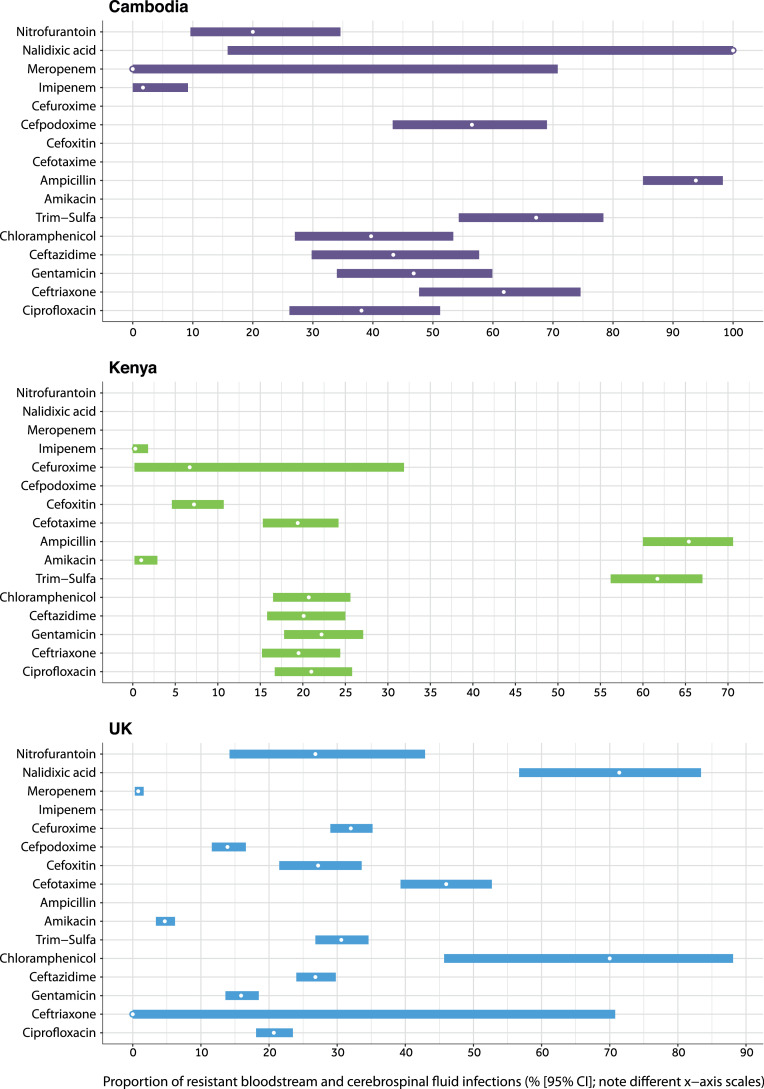
*Phenotypic resistance observed in Enterobacterales isolates causing bloodstream and cerebrospinal fluid infection in study settings.* Results are displayed for 16 antibiotics with susceptibility data across ≥ 2 settings from 2010 to 2017. Percentages are shown with 95% exact binomial confidence intervals (CI). “Trim-sulfa” is trimethoprim-sulfamethoxazole.

Phenotypic resistance in *Enterobacterales* isolates causing blood and cerebrospinal fluid infections was analysed for 16 antibiotics with antibiotic susceptibility test (AST) data in ≥2 study settings (Fig. 4). In Cambodia, resistance prevalence >30% was observed for all antibiotics except nitrofurantoin (not used to treat bloodstream or cerebrospinal fluid infections) and carbapenems; less phenotypic resistance was seen in isolates from Kenya and the UK (Fig. 4).

### 3.6 Modelling the prevalence of AMR in clinical infections from pooled faecal metagenomic data

**Fig. 5:**
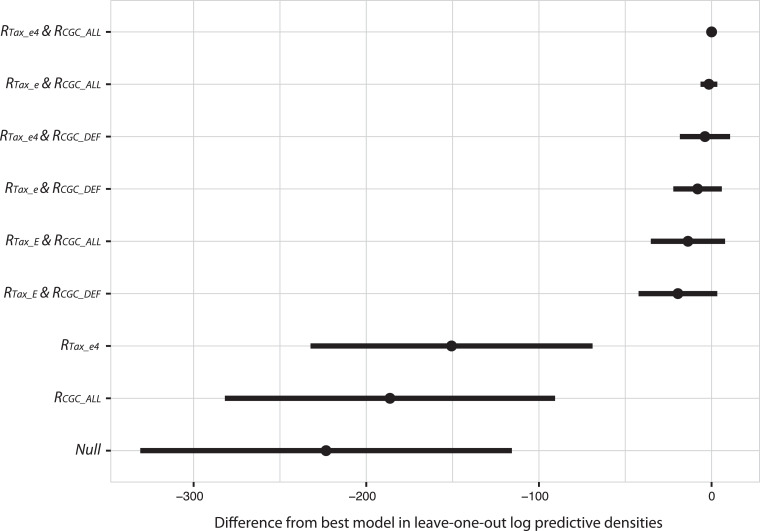
*Bayesian model comparison using leave-one-out cross-validation.* The leave-one-out prediction accuracy is shown on the *x*-axis measured as expected log pointwise predictive density [23] (elpd_loo) compared to the best performing model. The points show mean estimates and horizontal bars two times the standard error. The models are ordered from top to bottom by their mean elpd_loo difference to the best model. The best model is the model using *R_Tax_e4_* and *R_CGC_ALL_*.

The best taxonomy-adjusted AMR metric (resulting in the highest point-wise out of sample prediction accuracy based on cross-validation) used the taxonomic metric *R_Tax_e4_* measuring the commonest *Enterobacterales* species in clinical isolates (*Escherichia coli, Klebsiella pneumoniae, Salmonella* spp., *Enterobacter* spp.), and the abundance of AMR genes increasing the MIC or conferring clinically relevant resistance (*R_CGC_ALL_*). The model considering both *R_Tax_e4_* and *R_CGC_ALL_* as predictors outperformed other models in terms of out-of-sample predictions (Fig. 5). Models considering only AMR abundance (*R_CGC_ALL_*), or only taxonomic information (*R_Tax_e4_*) only marginally improved predictions relative to a baseline (null) model without any metagenomic information, whilst models with combined *R_CGC_* and *R_Tax_* showed substantially improved performance (difference in leave one out cross-validation log predictive densities compared to best model: Null [no *R_CGC_* and no *R_Tax_*] = −223 [−330,−116]; Baseline [*R_CGC_ALL_* only] = −186 [−281,−91]; Baseline [*R_Tax_e4_* only] = −151 [−232,−69]; appendix p14). Thus, we expect our best model considering both *R_Tax_e4_* and *R_CGC_ALL_,* to make substantially improved predictions compared to models with only one metric or no metagenomic metrics.

**Fig. 6:**
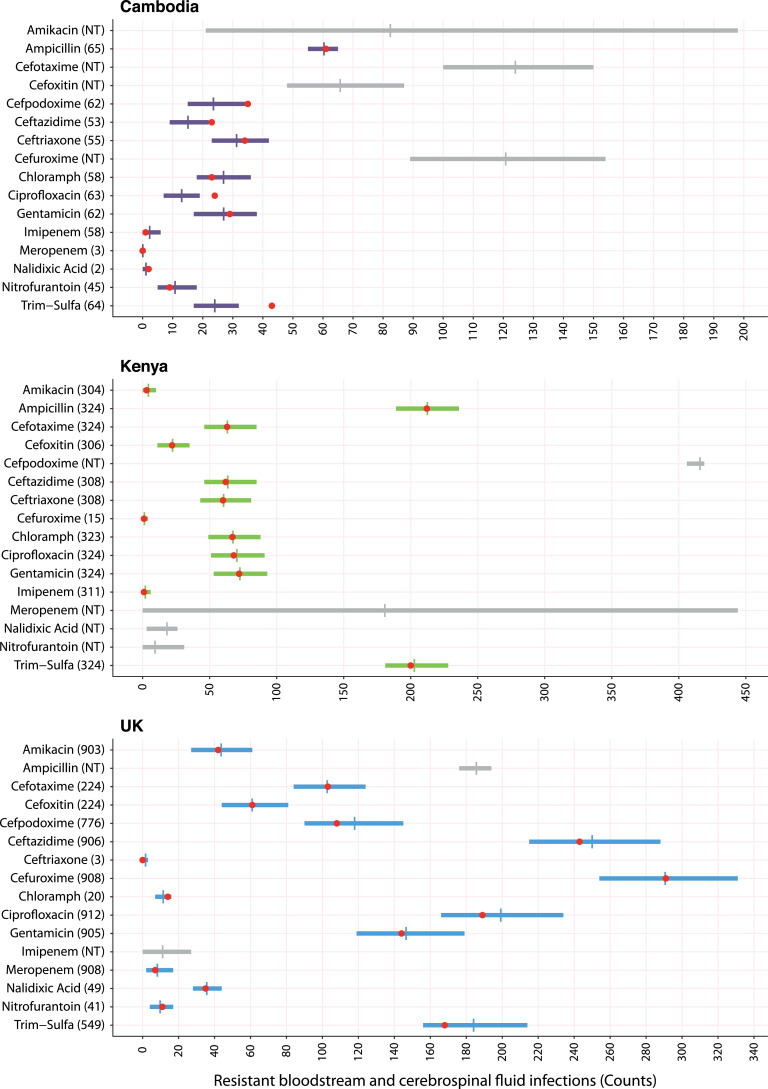
*Bayesian model prediction of resistant Enterobacterales bloodstream and cerebrospinal fluid infections in study settings.* Only antibiotics with antibiotic susceptibility test (AST) results in ≥2 settings are considered. Horizontal bars represent 95% highest density posterior interval and vertical lines represent means of the predicted resistant sample counts based on the model using metagenomic data from population pools. Coloured bars are shown where clinical data on resistance (i.e. AST) was available and grey bars where it was not. For grey bars the sample size was imputed. Red circles show the number of blood and cerebrospinal fluid *Enterobacterales* infections that were found to be resistant to the antibiotic listed in the *y*-axis. The number of isolates with AST results for each antibiotic are also given on the *y*-axis. Red circles are missing where no AST results were available. In cases where there is minimal uncertainty in the model estimate, the red circle may overshadow the 95% credible interval bars (e.g. meropenem [Cambodia]; cefuroxime [Kenya]). “Trim-sulfa” is short for trimethoprim-sulfamethoxazole; “Cloramph” is short for chloramphenicol. NT = no AST data available (For interpretation of the references to color in this figure legend, the reader is referred to the web version of this article.).

We compare the fits of the different models using logarithmic scoring, where a higher score indicates a better fit. The best model (*R_Tax_e4_* and *R_CGC_ALL_*) had a score of −133 (95% credible interval: −143, −123) and the null model a score of −298 (95% CI −304, −292). As a means to visualise the predictions of our best model, predictions were made for the 16 antibiotics with AST data in ≥2 settings for *Enterobacterales* isolates causing infection and then plotted against the observed resistant counts (Fig. 6). Predictions from the best model and those from the baseline (null) model are also presented in a table alongside the observed resistant counts for comparison purposes (appendix pp15-16). The mean-squared errors of the mean model predictions relative to the observations showed that the null model had an error of 468 compared to the best model, which had an error of 33. Bayesian model predictions expressed as percentages instead of counts are shown in appendix p17 for antibiotics with AST results from >100 invasive infection isolates (i.e. 14 antibiotics in the UK and/or Kenya).

## 4 Discussion

In this exploratory study we suggest that metagenomic analysis of pooled extracts from individual faecal samples could be effective at predicting resistance in invasive *Enterobacterales* infections from different age groups and geographic settings at the population-level, if both AMR gene abundances and taxonomy metrics from the pooled metagenomes are considered. Our approach would enable intermittent, relatively non-invasive sampling of a small subset of individuals within a population (e.g. 100–200), with a single centralised infrastructure (either in-country or internationally) undertaking metagenomic sequencing, analysis and prediction of population-level AMR in clinical *Enterobacterales* isolates. Although this could be done in parallel with the local development of microbiology laboratory networks, our approach would not be dependant on consistent sampling of individuals with infection. Our findings are supported by other studies successfully using sewage for global AMR surveillance and prediction of AMR in clinical isolates [8–10], but our approach could be more feasible in many LMIC settings where wastewater treatment/sewage infrastructures are scarce, and sewage sampling would therefore not be feasible. Population-level sampling could also overcome some of the potential biases affecting AMR prevalence estimates if only unwell individuals presenting to tertiary referral centres are sampled.

Based on pool size and sequencing depth (50–55 Gbp/pool), we avoided the need for potentially more expensive and labour-intensive individual indexing of DNA extracts in a pool, or the issues associated with targeted sequencing based on predefined AMR gene panels. Our strategy also enabled us to include samples with low DNA yields which may otherwise have failed library preparation; exclusion of these samples could potentially introduce bias. Uniquely, our bioinformatics pipeline incorporates the capacity to identify both specific AMR gene variants (e.g. *bla*_CTX__-__M-33_ versus *bla*_CTX__-__M-63_) alongside being able to aggregate by gene family. This is especially important as genes that differ by only single nucleotides/amino acids can have distinct phenotypic spectra. Although further validation of sample sizing and pooling strategies are needed, population pools comprising rectal swabs with as little as ≥1 ng/ul DNA/sample appeared to be sufficient to demonstrate predictive value in this proof-of-principle study.

Limitations of our approach were most obvious for the Cambodian setting, where observed resistance values from invasive isolates were within the 95% credible intervals of the best model predictions for only 75% of antibiotics. One explanation might be that the population pool erroneously included 19 additional longitudinal samples (12% of all samples in the pool) collected from neonates after hospitalisation, potentially compromising the analysis designed to reflect community-associated profiles (rapid changes occur in the neonatal resistome following hospitalisation/antibiotic exposures [32], and so this group may need more regular metagenomic sampling to accurately capture more rapid microbiome/resistome shifts). Cambodia was also the only setting where the age group in the metagenomics analysis (i.e. neonates), did not correspond exactly with the infection metadata analysed (i.e. infants ≤90 days of age). Clinical AST data in this setting were also scarce; the maximum number of isolates with AST results for any antibiotic was 65, compared to 324 in Kenya and 912 in UK. Ideally AST approaches would have been standardised across the settings. Finally, our analyses are heavily dependant on the accuracy of genotypic-phenotypic associations in the reference AMR gene catalogue. In general, however, we would expect this knowledge base to become increasingly robust, thus strengthening predictions. Our approach cannot be used for individual-level predictions; the value of accurate and rapid infectious diseases diagnostics in the management of individual patients remains clear.

Notwithstanding these limitations, we were able to predict AMR in clinical *Enterobacterales* isolates at the population-level using models that included AMR gene abundances and taxonomy metrics from the pooled faecal metagenomes, in three distinct geographic settings and age groups, in this exploratory study. We used a cross-validation approach to compare the prediction accuracy of the different models. It is notable that although no setting-specific parameters were included in the models, predictions from the best model in most cases showed good agreement with observed counts of resistant infections in each setting. Further studies to validate these promising proof-of-principle observations in additional settings across age categories, regions and in community versus healthcare-associated contexts, are warranted. There is potential to extend the approach to other priority bacteria and different colonisation samples. Future studies should also consider additional methodological simplification such as pooling all samples prior to DNA extraction. A mathematical framework for minimum-cost implementation of pooled-sample metagenomics-based surveys to quantify the burden of AMR in new settings without prior microbiology or AST data would also be of benefit, and could be greatly informed by the data we have generated.

Surveillance based on population colonisation metagenomics and taxonomy-adjusted AMR metrics presented here is a potentially valuable public health opportunity. This approach could theoretically be used to rapidly overcome the current paucity of quality AMR surveillance data and inform setting and population-tailored rationalization of empirical antibiotic use and treatment guidelines, develop measures to prevent and/or mitigate AMR, and ultimately improve public-health decision-making in conjunction with relevant stakeholders, especially in LMICs.

## Data sharing

The raw sequence data reported in this study have been deposited in the European Nucleotide Archive under accession number PRJEB34871. The code to extract CARD data, including relationship ontology terms that were required to generate the final datasets, plus any required input files, are available from the ResPipe GitLab repository (https://gitlab.com/hsgweon/ResPipe). This includes all commands and parameters run for with TrimGalore, Kraken2, Bracken, BBPMAP and ResPipe (the bioinformatics pipeline). The curated analysis datasets (Corrected gene counts; AMR__DEF_; AMR__ALL_; dataset for Bayesian analysis) can be found at https://data.mendeley.com/datasets/sxn6sw4r57/1 (Mendeley Data, V1, doi: 10.17632/sxn6sw4r57.1) along with the R code used to produce these and the code to run the Bayesian analysis.

## Funding

The study was funded by Bill & Melinda Gates Foundation (grant agreement OPP1160974) and was sponsored by University of Oxford. The study was also supported by the National Institute for Health Research (NIHR) Oxford Biomedical Research Centre, and NIHR Health Protection Research Unit in Healthcare-associated Infections and Antimicrobial Resistance (a partnership between the University of Oxford and Public Health England [PHE]). Kenyan samples were collected in a study funded by the MRC/DfID/Wellcome Joint Clinical Trials scheme: MR/M007367/1.

## Authors’ contributions

This work was first conceived by O.T.A., with support from N.S. and B.S.C.; O.T.A, N.S., B.S.C., R.N. and H.S.G. designed the study. K.C., J.W., O.T.A. and N.S. developed and validated modified DNA extraction protocols for this study. K.C., J.W. and R.B. conducted or facilitated most of the pre-sample-pooling laboratory work.  S.L. designed the methods and provided technical guidance for sample pooling and sequencing and conducted the sequencing work. J.A.B., J.D.E., P.T. and R.B. facilitated the collation and transfer of samples and data from participant settings. They also provided technical support for clinical and microbiology study procedures and for the development of context-appropriate standard operating procedures. N.S., A.S.W., T.E.P., D.W.C. and B.S.C. provided support and guidance for all technical aspects of the study (including for bioinformatics and data analyses) and contributed to the revision of study outputs. T.N. contributed to the mining, standardisation and analysis of infection metadata from each setting. H.S.G. conducted the bioinformatics work, designed the methods for corrected gene counts and extracted the data from CARD. J.S. provided the computing support for the study. O.T.A., H.S.G conducted mining, linkage and visualisation of study data. R.N. conducted the validation and Bayesian analyses and B.S.C. contributed to revision of these methods. O.T.A, N.S., R.N. and H.S.G. produced the first manuscript draft. All authors contributed significantly to the iterative review of the draft. O.T.A, N.S., R.N., H.S.G., J.A.B., P.T. and R.B verified the underlying data for this article.

## Declaration of Competing Interest

The authors declare no competing interests.

## Appendix Supplementary materials

Image, application 1

Image, application 2

Image, application 3

Image, application 4

Image, application 5
