# Supplementary material for: Population-level faecal metagenomic profiling as a tool to predict antimicrobial resistance in Enterobacterales isolates causing invasive infections: An exploratory study across Cambodia, Kenya, and the UK
Source: eClinicalMedicine. 2021 May 30;36:100910. doi: 10.1016/j.eclinm.2021.100910 (PMC8173267; doi:10.1016/j.eclinm.2021.100910)
Supplement: Supplementary file 1 [file mmc1.pdf]

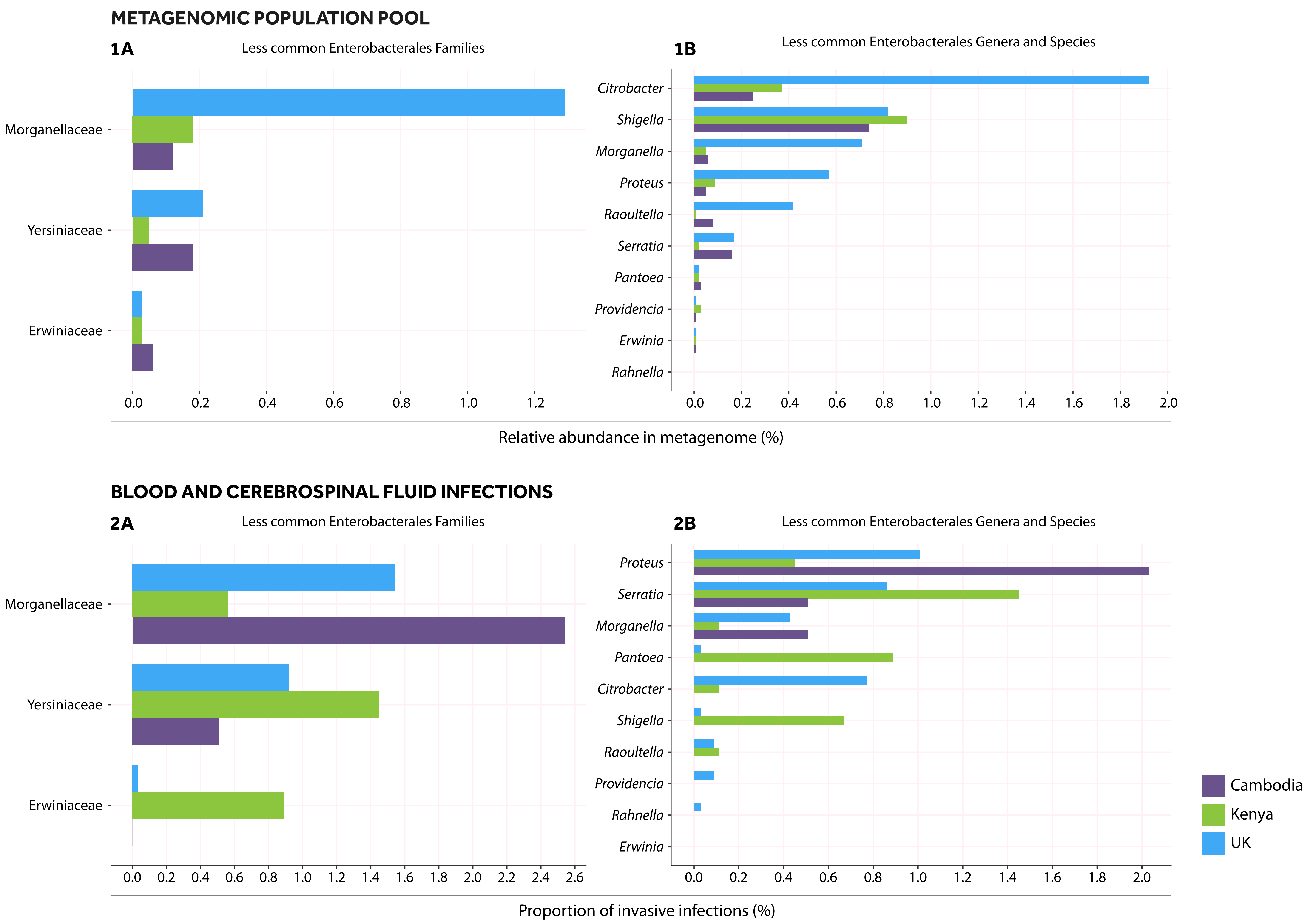
METAGENOMIC POPULATION POOL
1A
Less common Enterobacterales Families
Morganellaceae
Yersiniaceae
Erwiniaceae
0.0
0.2
0.4
0.6
0.8
1.0
1.2
1B
Less common Enterobacterales Genera and Species
Citrobacter
Shigella
Morganella
Proteus
Raoultella
Serratia
Pantoea
Providencia
Erwinia
Rahnella
0.0
0.2
0.4
0.6
0.8
1.0
1.2
1.4
1.6
1.8
2.0
Relative abundance in metagenome (%)
BLOOD AND CEREBROSPINAL FLUID INFECTIONS
2A
Less common Enterobacterales Families
Morganellaceae
Yersiniaceae
Erwiniaceae
0.0
0.2
0.4
0.6
0.8
1.0
1.2
1.4
1.6
1.8
2.0
2.2
2.4
2.6
2B
Less common Enterobacterales Genera and Species
Proteus
Serratia
Morganella
Pantoea
Citrobacter
Shigella
Raoultella
Providencia
Rahnella
Erwinia
0.0
0.2
0.4
0.6
0.8
1.0
1.2
1.4
1.6
1.8
2.0
Proportion of invasive infections (%)
Cambodia
Kenya
UK
