# Supplementary figures and images for: Population-level faecal metagenomic profiling as a tool to predict antimicrobial resistance in Enterobacterales isolates causing invasive infections: An exploratory study across Cambodia, Kenya, and the UK
Source: eClinicalMedicine. 2021 May 30;36:100910. doi: 10.1016/j.eclinm.2021.100910 (PMC8173267; doi:10.1016/j.eclinm.2021.100910)

All Genera

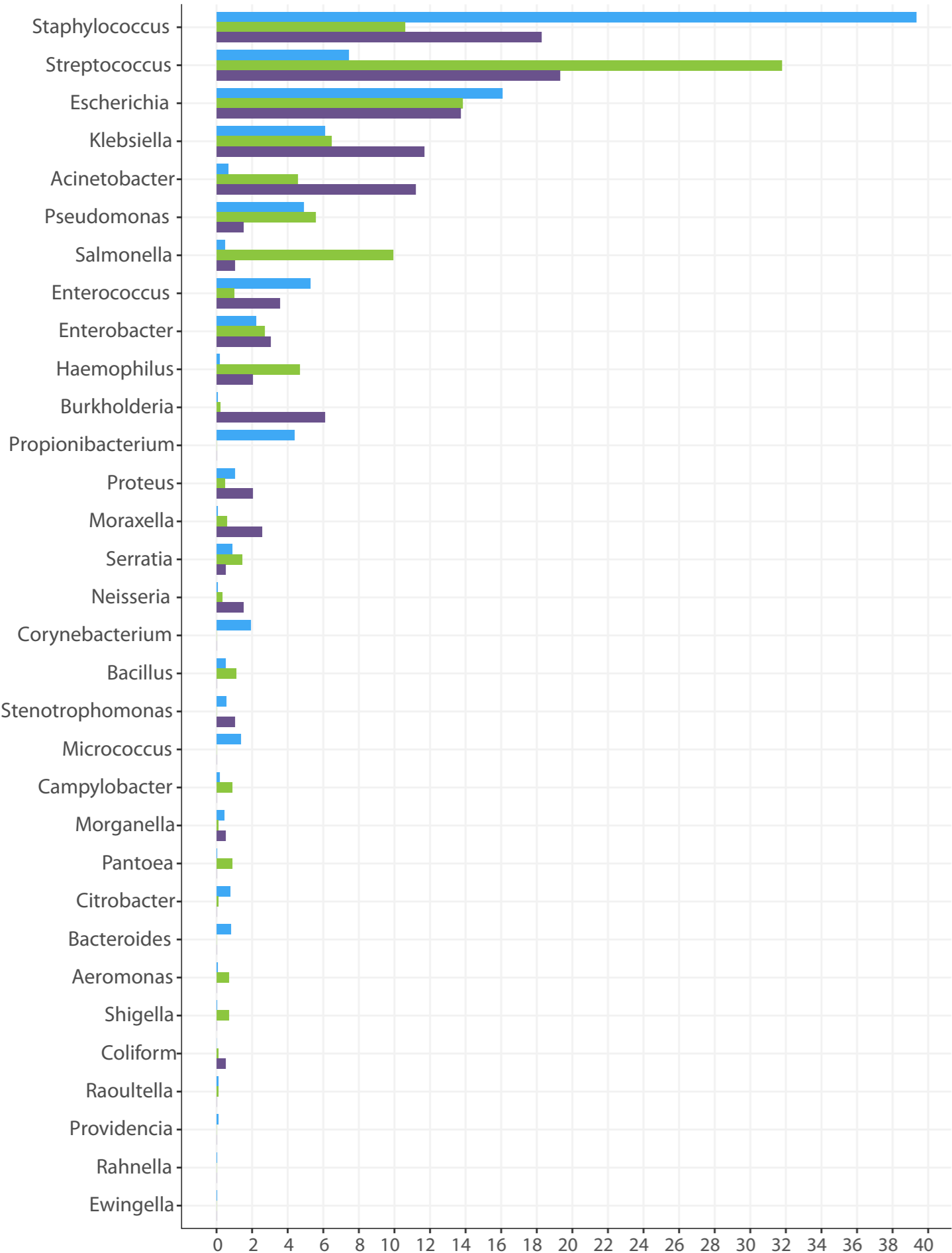

All Species

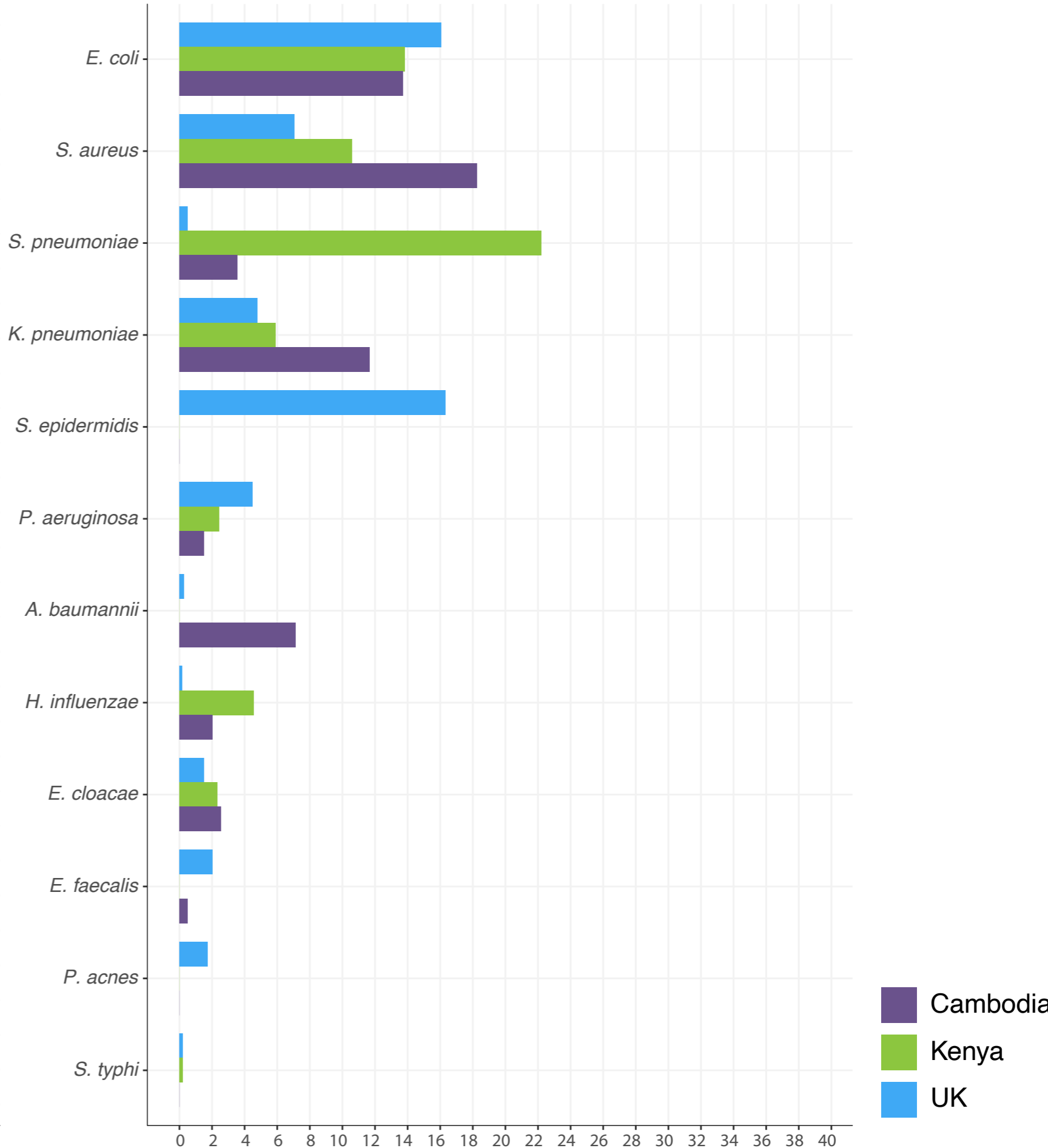

Bloodstream and cerebrospinal fluid infections (%)

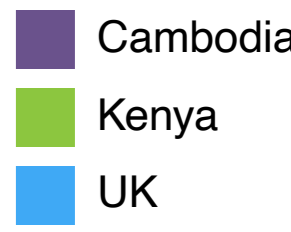

Supplement: Supplementary file 2 [file mmc2.pdf]

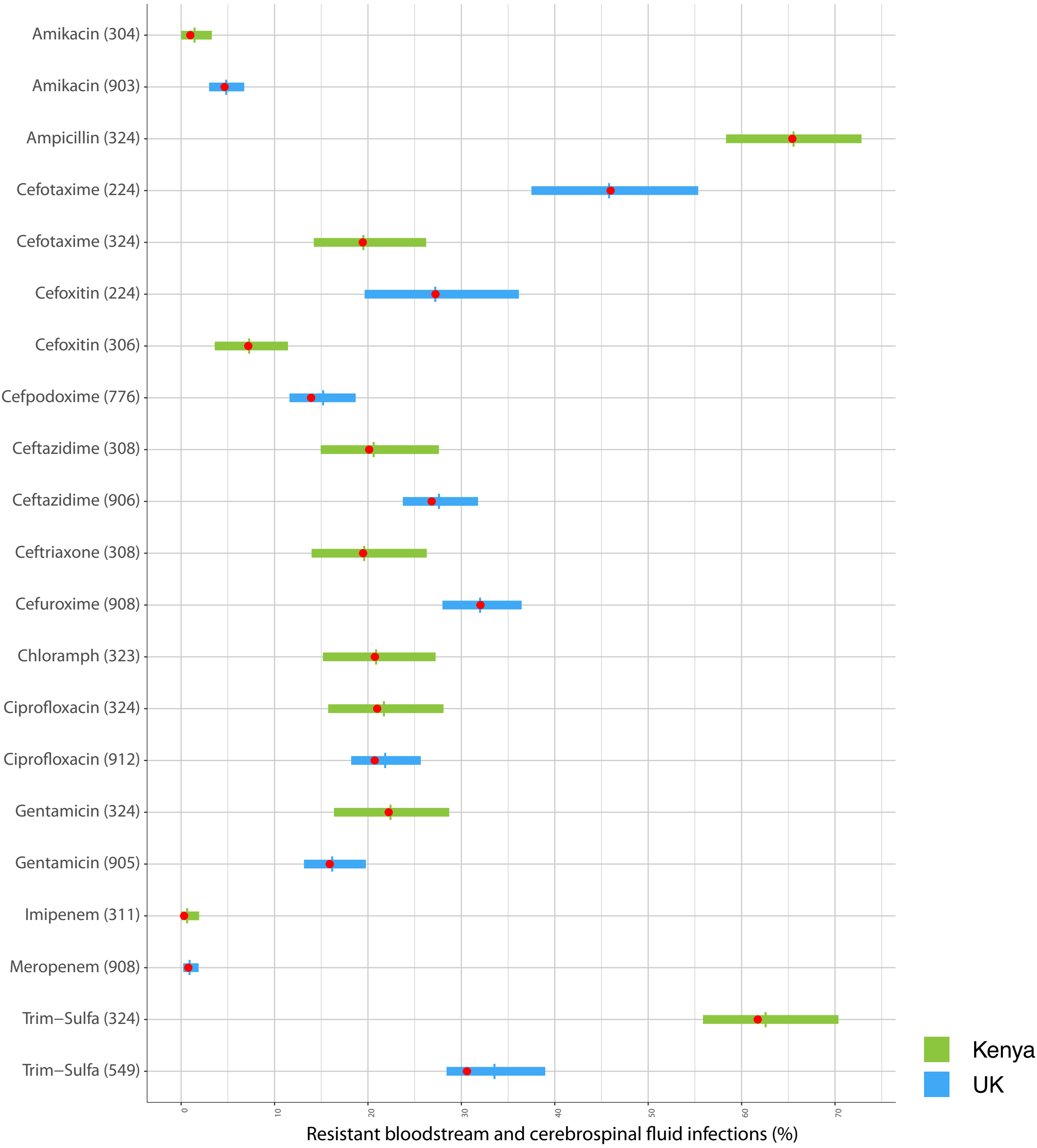

Supplement: Supplementary file 3 [file mmc3.pdf]

**A. CORRECTED RESISTANCE GENE COUNTS (CGCs)**

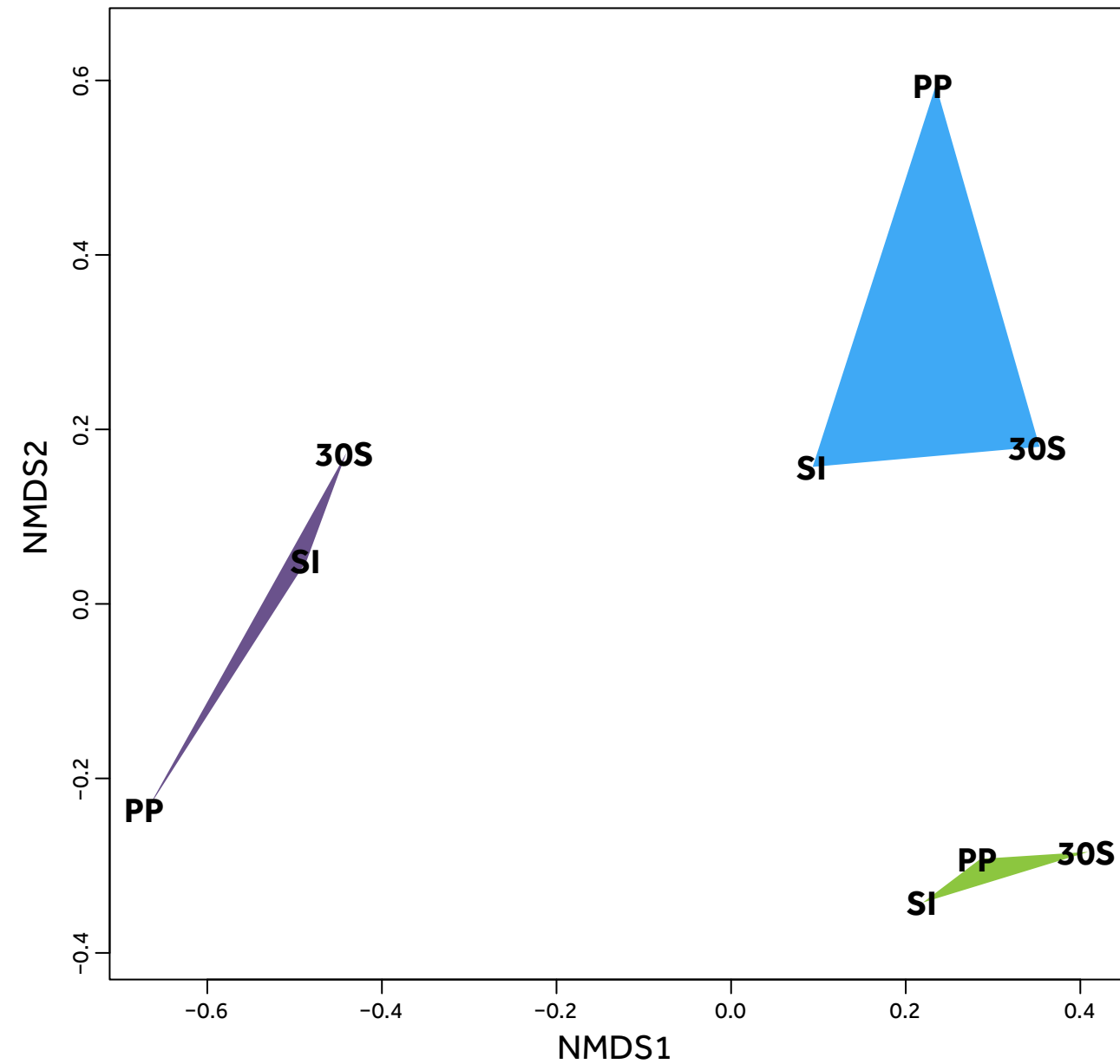

**B. RELATIVE ABUNDANCE OF RESISTANCE GENES**

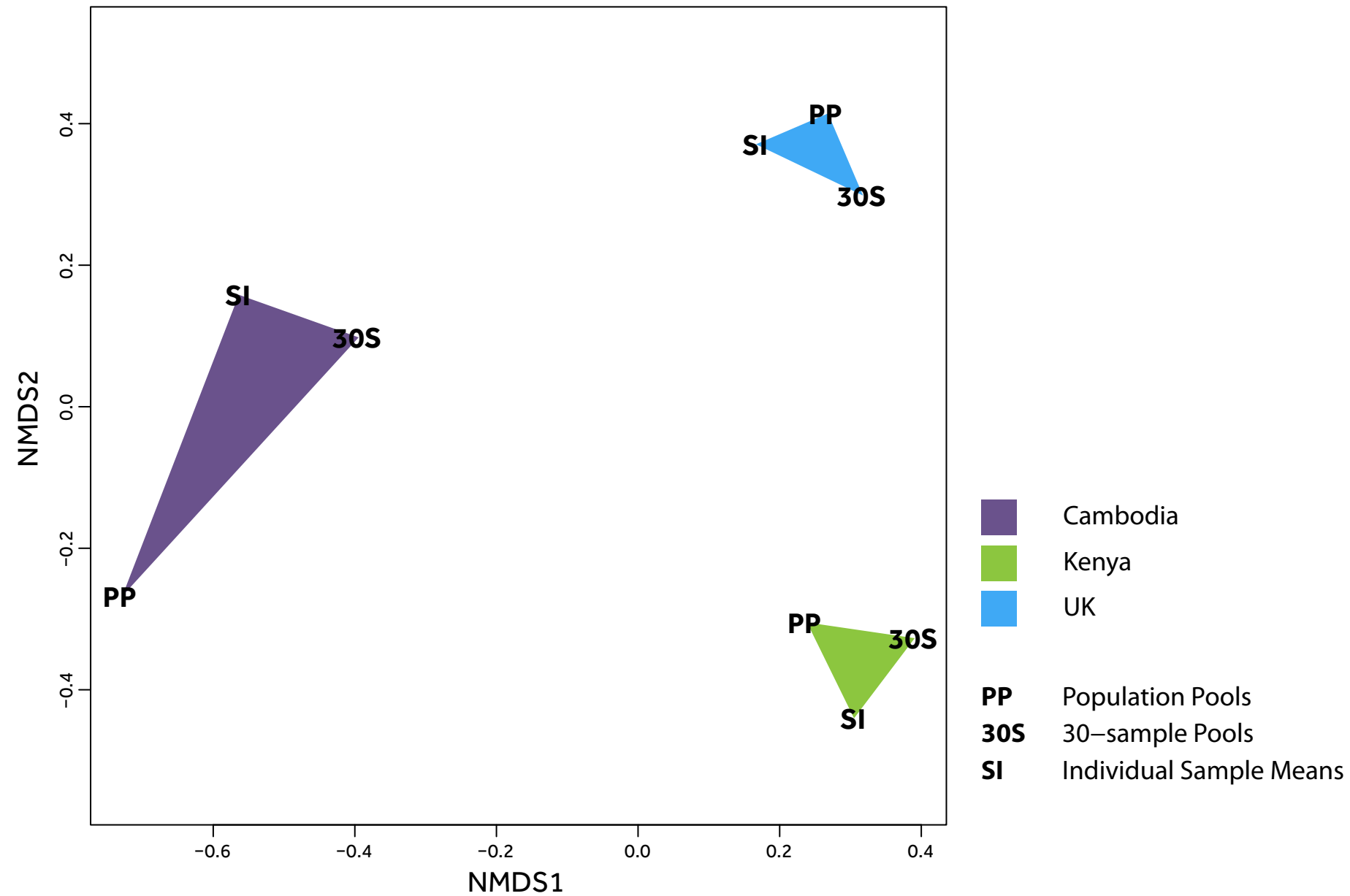

Supplement: Supplementary file 4 [file mmc4.pdf]
