# Supplementary material for: Population-level faecal metagenomic profiling as a tool to predict antimicrobial resistance in Enterobacterales isolates causing invasive infections: An exploratory study across Cambodia, Kenya, and the UK
Source: eClinicalMedicine. 2021 May 30;36:100910. doi: 10.1016/j.eclinm.2021.100910 (PMC8173267; doi:10.1016/j.eclinm.2021.100910)
Supplement: Supplementary file 5 [file mmc5.docx]

**Supplementary appendix**

**Table of Contents**

[1. Supplementary methods 2](#_Toc67039532)

[1.1 Samples and settings 2](#_Toc67039533)

[1.2 Invasive infection metadata 2](#_Toc67039534)

[1.3 DNA extraction 4](#_Toc67039535)

[1.4 Sample pooling and metagenomic sequencing 9](#_Toc67039536)

[1.5 Sequence data processing 9](#_Toc67039537)

[1.6 Bayesian modelling 9](#_Toc67039538)

[1.7 Validation of pooling 11](#_Toc67039539)

[2 Supplementary results 12](#_Toc67039540)

[2.1 Less common *Enterobacterales* in metagenomic population pools and in bloodstream and cerebrospinal fluid infections. 12](#_Toc67039541)

[2.2 Main species and genera (all bacterial orders) identified from bloodstream and cerebrospinal fluid infections. 13](#_Toc67039542)

[2.3 Bayesian model comparisons 14](#_Toc67039543)

[2.4 Model predictions for the best model, compared to the null model 15](#_Toc67039544)

[2.5 Bayesian model prediction of proportion of *Enterobacterales* invasive infections with resistance to antibiotics 17](#_Toc67039545)

[2.6 Pooling faecal DNA extracts as a strategy to measure population-level AMR gene distributions 18](#_Toc67039546)

[3 References 20](#_Toc67039547)

# **Supplementary methods**

## **Samples and settings**

Faecal material from existing biobanks was chosen for study. Samples comprised:

1. Rectal swabs from children aged 1-59 months, taken on admission to Kilifi County Hospital in Kilifi, Kenya, from 1^st^ April to 30^th^ September 2016. These samples were stored in Amies transport media + 1ml phosphate buffered saline at -80°C;
2. Faecal samples taken from newborns on admission to Angkor Hospital for Children in Siem Reap, Cambodia, from 11^th^ September 2013 to 10^th^ September 2014, and stored in tryptone soya broth + 10% glycerol at -80°C. This collection also included longitudinal samples taken from a subset of newborns during their inpatient stay for another study; and
3. Rectal swabs (Eswab, Copan diagnostics, Murrieta, CA, USA; with 1 ml of liquid Amies) from individuals aged ≥18 years attending pre-admission clinics or on admission to Guy’s and St Thomas’ NHS Foundation Trust, London, UK, between February and May 2015, and stored at -80°C.

These samples were collected as part of the following studies:

1. The ‘*Pharmacokinetics of Antimicrobials and Carriage of Antimicrobial Resistance amongst Hospitalised Children with Severe Acute Malnutrition (FLACSAM)*’ study in Kenya^1^ (ethics approval references: KEMRI/SERU/CGMR- C/023/3161; OXTREC 47-15);
2. The ‘*Colonisation of a new neonatal unit: defining organisms, rates and transmission pathways*’ study in Cambodia^2^ (ethics approval reference: OxTREC ref 1047-13; this collection also included longitudinal samples taken from a subset of newborns during their inpatient stay for another study);
3. The ‘*Enhanced surveillance for resistant gram-negative bacteria*’ study in UK^3–5^ (ethics approval reference: REC - 14/LO/2085).

## **Invasive infection metadata**

The SOP for collection of invasive infection metadata from each study site is given in box S1. Each site has a microbiology laboratory participating in external quality assurance schemes (e.g. UK National External Quality Assessment Service, NEQAS; WHO EQA scheme supported by the Cambodian Ministry of Health) and is additionally accredited to UK ISO15189 (London laboratory; underway in Cambodia) or WHO Good Clinical Laboratory Practice standards (Kilifi laboratory). Catchment areas served by each laboratory vary: For Cambodia about two-thirds of the patients come from within Siem Reap province^6,7^; in Kenya the population served is mostly rural, within the coastal Kilifi District^8^; and in London the laboratory largely serves a South London community of approximately 0.5 million people and also regularly provides services to international patients and patients from other sites in the UK^9^. Collated metadata included bacterial species identification results, available antibiotic susceptibility test (AST) results, specimen type and basic patient details to validate aggregate-level stratification by age. Samples were processed using standard operating procedures in accordance with accredited guidelines. In the UK, the VITEK system (bioMérieux, Marcy-l'Etoile, France) was used for AST and performed according to the British Society for Antimicrobial Chemotherapy standards^10^ (BSAC). In Cambodia and Kenya, AST was performed using a standardised disk diffusion method following the Clinical and Laboratory Standards Institute (CLSI) guidelines^11^. Where accurate AST results could not be achieved by simple disk diffusion, minimum inhibitory concentrations (MICs) were determined by Etest in both settings. The infection metadata was collated for infants < 90 days of age in Cambodia, ≤ 60 months of age in Kenya and ≥ 18 years of age in the UK.

**Box S1 Short SOP for collation of infection metadata from each study site**

| Aim: to collate raw data at the individual-patient level, and to aggregate the data to address the objectives of the research.   1. **Inclusion Criteria:**     1. Date range:       1. From 01/01/2010 to 31/05/2017    2. Specimen types: Invasive bacterial isolates from       1. Blood cultures       2. Cerebrospinal fluid samples    3. Age groups:       1. 0-60 months of age (Kenya)       2. 0-89 days of age (Cambodia)       3. ≥ 18 years of age (UK)    4. Patient-sample type:       1. Inpatient samples on admission (i.e. obtained 0-72 hours from admission) 2. **Basic Fields:**     1. Anonymised patient identifier       1. e.g. CamPat_01; CamPat_02…       2. e.g. KenPat_01; KenPat_02…       3. e.g. LonPat_01; LonPat_02…    2. Patient’s age       1. Report age in days (from DOB to sample date [Kenya and Cambodia])       2. Report age in years (from DOB to sample date; round up to the lowest number [e.g. 25.1 = 25 years; 25.9 = 25 years; UK])    3. Patient’s gender       1. M/F    4. Admission date       1. dd/mm/yyyy    5. Sample date       1. dd/mm/yyyy    6. Anonymised specimen identifier       1. e.g. CamSP_01; CamSP_02…       2. e.g. KenSP_01; KenSP_02…       3. e.g. LonSP_01; LonSP_02…    7. Specimen type       1. CSF       2. Blood culture    8. Bacterial speciation results       1. Each bacterial species/genus to be placed on a separate row. Aim to provide detailed data (i.e. species preferred to genus, etc.)    9. Complete antibiotic susceptibility test (AST) results       1. Place each antibiotic on a separate column          1. Report disk diffusion zone (DDZ) or minimum inhibitory concentration (MIC), specifying units in column heading          2. If/where DDZ or MIC is not available, provide column detailing resistance as R (resistant) / I (intermediate) / S (susceptible). See additional notes below. 3. **Additional fields (where feasible):**    1. Previous admission to reporting hospital       1. No previous admission within the past ≤ 12 months (Code=0)       2. Previous admission within > 6 and ≤ 12 months (Code=1)       3. Previous admission within ≤ 6 months (Code=2)    2. Severe Acute Malnutrition (Kenya)       1. Y/N    3. Ethnicity (UK) 4. **Notes:**    1. Code all missing data as ‘NA’.    2. Provide outline methodology for DST and cut-off points for classification of S/R/I if/where appropriate. Please detail any changes in DST testing over the study period (e.g. including addition and/or removal of antibiotics from the tested battery over time).    3. Report all admissions from a same patient over time (i.e. not only the first admission from a same patient over the study period) |
| --- |

## **DNA extraction**

Samples from Cambodia and Kenya were shipped to the Nuffield Department of Medicine (University of Oxford, UK) for extraction; extractions for London samples took place at the Centre for Clinical Infection and Diagnostics Research (CIDR-King’s College London). Detailed methods on extractions from rectal swabs/faecal samples to achieve sufficient DNA yields for sequencing (ideally ≥300ng DNA/34ul, with a view to obtaining ≥20Gbp (Giga base pairs) of data per sample) are given in the modified DNA extraction protocols below (Protocol 1 & 2). Known copy numbers of internal standards consisting of *Thermus thermophilus* HB8 genomic DNA^12^ (not normally present in faecal samples) were added to each sample prior to the addition of Solution C1 (i.e. 8.75 ul per sample [1ng/ul of Thermus DNA]). The presence of *T. thermophilus* was ascertained following sequencing by mapping reads to the Thermus reference genome.

**Protocol 1 - Modified MoBio PowerSoil DNA extraction method for metagenomic DNA extraction from rectal swabs**

Rectal swabs suspended in Phosphate Buffer Saline (PBS) or Amies trasport media (ATM) and stored at -80°C, were thawed on ice. The MoBio PowerSoil DNA Isolation Kit (Qiagen, Hilden, Germany) was used for DNA extraction using the following modified SOP:

1. **Sample Preparation and Initial Lysing Steps**

Before getting started: Check Solution C1. If Solution C1 is precipitated, heat solution to 60°C until dissolved before use.

1. Transfer swab head into the PowerBead tube without vortexing the thawed sample.
2. Centrifuge the original sample tube, which now only contains PBS or ATM, at 13,200 rpm for 10 min.
3. Carefully remove the supernatant from the original sample tube (about 800ul of PBS or ATM) without disturbing the pellet.
4. Re-suspend the pellet using the remaining 200 μl of PBS or ATM in the sample tube.
5. Transfer the mix into the PowerBead tube.
6. Add *Thermus thermophilus*^[[1]](#footnote-1)^ DNA (8.75 ul/sample [1ng/ul]).
7. Add 60 μl of Solution C1 to the PowerBead tube.
8. Vortex for 5 seconds.
9. Bead beat^[[2]](#footnote-2)^ PowerBead tubes using the following set up: 6.0 m/s, 40 seconds, 2 cycles, 300 second pause, 1 ml volume, Lysing matrix E. Incubate rack and tubes at 4°C between cycles.
10. **DNA extraction**
11. Incubate PowerBead tubes at 65°C for 10 minutes.
12. Vortex for 5 seconds.
13. Incubate PowerBead tubes at 65°C for another 10 minutes.
14. Vortex for 5 seconds.
15. Incubate PowerBead tubes at 95°C for 10 minutes.
16. Incubate PowerBead tubes at 4°C for 5 minutes.
17. Bead beat^2^ PowerBead Tubes using the following set up: 6.0 m/s, 40 seconds, 2 cycles, 300 second pause, 1 ml volume, Lysing matrix E. Incubate rack and tubes at 4°C between cycles.
18. Centrifuge tubes at 10,000 x g for 30 seconds at room temperature. Make sure the PowerBead tubes rotate freely in your centrifuge without rubbing.

CAUTION: Be sure not to exceed 10,000 x g or tubes may break.

1. Transfer 600 μl of the supernatant to a clean 2 ml Collection Tube.
2. Add 250 μl of Solution C2.
3. Vortex for 5 seconds.
4. Incubate at 4°C for 5 minutes.
5. Centrifuge the tubes at room temperature for 2 minutes at 10,000 x g.
6. Avoiding the pellet, transfer up to, but no more than, 600 μl of supernatant to a clean 2 ml Collection Tube.
7. Add 200 μl of Solution C3.
8. Vortex briefly.
9. Incubate at 4°C for 5 minutes.
10. Centrifuge the tubes at room temperature for 1 minute at 10,000 x g.
11. Avoiding the pellet, transfer up to, but no more than, 750 μl of supernatant into a clean 2 ml Collection Tube.
12. Shake to mix Solution C4 before use.
13. Add 1200 μl of Solution C4 to the supernatant and vortex for 5 seconds.
14. Load approximately 675 μl onto a Spin Filter and centrifuge at 10,000x g for 1 minute at room temperature. Discard the flow through and add an additional 675 μl of supernatant to the Spin Filter and centrifuge at 10,000 x g for 1 minute at room temperature. Load the remaining supernatant onto the Spin Filter and centrifuge at 10,000 x g for 1 minute at room temperature.

NOTE: A total of three loads for each sample processed are required.

1. Add 500 μl of Solution C5 and centrifuge at room temperature for 30 seconds at 10,000 x g.
2. Discard the flow through.
3. Centrifuge again at room temperature for 1 minute at 10,000 x g.
4. Carefully place spin filter in a clean 2 ml Collection Tube. Avoid splashing any Solution C5 onto the Spin Filter.
5. Add 50 μl of Solution C6 to the centre of the white filter membrane.
6. Incubate Spin Filters at room temperature for 2 minutes.
7. Centrifuge at room temperature for 30 seconds at 10,000 x g.
8. Discard the Spin Filter. The DNA in the tube is now ready for any downstream application. No further steps are required.

**Protocol 2 - Modified MoBio PowerSoil DNA extraction method for metagenomic DNA extraction from faecal slurry samples**

Faecal slurry samples (faeces suspended in nutrient broth +10% glycerol) stored at -80°C, were thawed on ice. The MoBio PowerSoil DNA Isolation Kit (Qiagen., Hilden, Germany) was used for DNA extraction using the following modified SOP:

1. **Assess Pellet size**
2. Weigh and record empty microcentrifuge tube weights
3. Vortex thawed samples and transfer samples to pre-weighed microcentrifuge tubes using wide orifice pipette tips
4. Centrifuge at 13,200 rpm for 10 min
5. Carefully remove the supernatant without disturbing the pellet
6. Re-weigh microcentrifuge tube and calculate pellet weight
7. Resuspend the pellet using 500 μl of buffer from a PowerSoil® DNA Isolation Kit Bead tube using wide orifice pipette tips
8. Transfer the mix back into the Bead tube and proceed with DNA extraction or store at -80°C for future extraction
9. **DNA extraction**

Before getting started: Check Solution C1. If Solution C1 is precipitated, heat solution to 60°C until dissolved before use.

1. Add *Thermus thermophilus*^[[3]](#footnote-3)^ DNA (8.75 ul/sample [1ng/ul])
2. Vortex the PowerBead tube containing resuspended faecal pellet for 3 seconds.
3. Add 60 μl of Solution C1 and vortex for 5 seconds.
4. Incubate PowerBead Tubes at 65°C for 10 minutes.
5. Vortex for 5 seconds.
6. Incubate PowerBead Tubes at 65°C for another 10 minutes.
7. Vortex 5 seconds.
8. Incubate PowerBead Tubes at 95°C for 10 minutes.
9. Incubate PowerBead Tubes at 4°C for 5 minutes.
10. Bead Beat PowerBead Tubes using the following protocol: 6.0 m/s, 40 seconds, 2 cycles, 300 second pause, 1 ml volume, Lysing matrix E. Incubate rack and tubes at 4°C between cycles.
11. Make sure the PowerBead Tubes rotate freely in your centrifuge without rubbing. Centrifuge tubes at 10,000 x g for 30 seconds at room temperature.

CAUTION: Be sure not to exceed 10,000 x g or tubes may break.

1. Transfer 600 μl of the supernatant to a clean 2 ml Collection Tube.
2. Add 250 μl of Solution C2 and vortex for 5 seconds. Incubate at 4°C for 5 minutes.
3. Centrifuge the tubes at room temperature for 2 minutes at 10,000 x g.
4. Avoiding the pellet, transfer up to, but no more than, 600 μl of supernatant to a clean 2 ml Collection Tube.
5. Add 200 μl of Solution C3 and vortex briefly. Incubate at 4°C for 5 minutes.
6. Centrifuge the tubes at room temperature for 1 minute at 10,000 x g.
7. Avoiding the pellet, transfer up to, but no more than, 750 μl of supernatant into a clean 2 ml Collection Tube.
8. Shake to mix Solution C4 before use. Add 1200 μl of Solution C4 to the supernatant and vortex for 5 seconds.
9. Load approximately 675 μl onto a Spin Filter and centrifuge at 10,000x g for 1 minute at room temperature. Discard the flow through and add an additional 675 μl of supernatant to the Spin Filter and centrifuge at 10,000 x g for 1 minute at room temperature. Load the remaining supernatant onto the Spin Filter and centrifuge at 10,000 x g for 1 minute at room temperature. A total of three loads for each sample processed are required.
10. Add 500 μl of Solution C5 and centrifuge at room temperature for 30 seconds at 10,000 x g.
11. Discard the flow through.
12. Centrifuge again at room temperature for 1 minute at 10,000 x g.
13. Carefully place spin filter in a clean 2 ml Collection Tube. Avoid splashing any Solution C5 onto the Spin Filter.
14. Add 70 μl of Solution C6 to the centre of the white filter membrane.
15. Incubate Spin Filters at room temperature for 2 minutes.
16. Centrifuge at room temperature for 30 seconds at 10,000 x g.
17. Discard the Spin Filter. The DNA in the tube is now ready for any downstream application. No further steps are required.

## **Sample pooling and metagenomic sequencing**

Sequencing of all faecal sample DNA extracts (pools and individual extracts [i.e. 96 metagenomes comprising 90 individual metagenomes; three 30-sample pools; three population pools]) was performed using the HiSeq 4000 Illumina platform, at the Wellcome Trust Centre for Human Genetics, Oxford, UK.

500ng of DNA from each sample was used for library preparation. Libraries were constructed using the NEBNext Ultra DNA Sample Prep Master Mix Kit (NEB) with minor modifications and a custom automated protocol on a Biomek FX (Beckman)^13^. At the time of sequencing, the HiSeq 4000 produced on average 72-90 Gbp of data per lane. We sequenced four individual extracts per lane to obtain on average 18-22.5 Gbp of data per sample. For the pooled samples, we sequenced one 30-sample-pool plus one population-pool per lane to obtain on average 36-45 Gbp of data per pool. Metagenomic data was obtained once for each distinct sample or pool; there were no technical replicates due to the expense of high-throughput sequencing, but the replicability of this approach has been evaluated in a previous study^14^.

## **Sequence data processing**

The taxonomic abundance of bacterial species and AMR genes at individual and pooled sample levels was determined using a published bioinformatics pipeline, ResPipe^14^ (default parameters were used unless stated otherwise). Briefly, as part of the quality filtering, the sequenced paired-end reads were filtered based on PHRED scores and length (≥ Q25 and ≥ 50 bp), and adapters removed using TrimGalore^15^. For profiling the abundance of bacterial species, as part of ResPipe, the quality-filtered sequences were classified with Kraken2^16^ (v.2.0.8-beta) against bacteria, plasmid, viral and human genome sequences recovered from NCBI (12 July 2019). With the taxonomic classification from Kraken2 and information about species specific versus non-specific genetic regions we estimated abundances at the species level using Bracken^17^ (v.2.5.0), which was subsequently used to derive relative abundances of bacterial taxa. Again as part of ResPipe’s AMR gene profiling, quality-filtered sequences were mapped against the Comprehensive Antibiotic Resistance Database^18,19^ (CARD, v.3.0.3) using BBMAP^20^ (v.37.72) at 100% sequence identity. All AMR genes identified in any of the samples were included in the analysis. The number of sequences mapping to each AMR gene was corrected to remove reference gene length bias. This was done using four metrics, namely: (1) specific read count (number of sequences mapping exclusively to the reference AMR gene); (2) specific lateral coverage (proportion of the AMR gene covered by sequences mapping exclusively to the gene); (3) AMR gene length; and (4) and average read length (average length of reads that mapped to the AMR gene), using the following formula: corrected gene count (CGC)=(specific read count x average read length)/(AMR gene length x specific lateral coverage).

AMR diversity (richness) and dissimilarity between sample types (non-metric multidimensional scaling [NMDS]) were derived from rarefied sequencing data where (1) both 30-sample-pools and population-pools samples were rarefied to 168,688,560 sequences; (2) each individual sample was first rarefied to 5,622,952 sequence and subsequently pooled to 168,688,560 sequences. This was to ensure that individual samples were equally weighted, and the sampling efforts/depths were the same across all sample types in the three different settings (i.e. 30-sample-pool, population-pool and individual samples). For comparing relative abundances of AMR gene profiles and modelling, we used a relative abundance metric, *R_CGC_*, defined as the sum of corrected gene counts (CGCs) of AMR gene variants associated with resistance to a given antibiotic, *j (R_CGCj_*), divided by the total CGC of all AMR genes in the pool. Do note that *R_CGC_*, was derived from unrarefied sequencing data given that the metric is for comparing relative abundances of AMR genes rather than diversity.

## **Bayesian modelling**

For each of the six taxonomy-adjusted AMR metrics (table S1), we fitted a Bayesian generalized linear model to the infection data and compared models in terms of their out of sample prediction accuracy, using leave-one-out cross-validation^21^ (section 2.3). This was used to determine the best taxonomy-adjusted AMR metric. In addition, we also assessed for the best model and our baseline (null) model the within sample predictions of clinical invasive *Enterobacterales* isolates from each setting (section 2.4).

**Table S1 Bayesian models**

| **Models with taxonomy-adjusted AMR metrics** | **Metric Combinations** | |
| --- | --- | --- |
|  | ***R_Tax_*** | ***R_CGC_*** |
| 1 | *R_Tax_E_* | *R_CGC_ALL_* |
| 2 | *R_Tax_e_* | *R_CGC_DEF_* |
| 3 | *R_Tax_e4_* | *R_CGC_ALL_* |
| 4 | *R_Tax_E_* | *R_CGC_DEF_* |
| 5 | *R_Tax_e_* | *R_CGC_ALL_* |
| 6 | *R_Tax_e4_* | *R_CGC_DEF_* |
| **Baseline Models** |  |  |
| 1 (Null) | *-* | *-* |
| 2 | *-* | *R_CGC_* |
| 3 | *R_Tax_* |  |

*R_Tax_E_: Enterobacterales* / Total bacterial taxa in the pool; *R_Tax_e_: Enterobacteriaceae* / Total bacterial taxa in the pool; *R_Tax_e4_:* Combined abundance of *Escherichia coli*, *Klebsiella pneumoniae*, *Salmonella spp* and *Enterobacter spp* / Total bacterial taxa in the pool. *R_CGC_ALL:_ R_CGC_ALLj_ /* Total *R_CGC_ALL_* in the pool; *R_CGC_DEF:_ R_CGC_DEFj_ /* Total *R_CGC_DEF_* in the pool. Whichever *R_Tax_* and *R_CGC_* metric performed best in models considering combinations (models 1-6), was then considered in baseline models including only *R_CGC_* (baseline model 2) or only *R_Tax_* (baseline model 3).

We let *i* denote the setting (Cambodia, Kenya or UK), and *j* the antibiotic being evaluated (see below for a complete list of parameters). We assumed that counts of resistant samples follow a binomial distribution. Our model then predicts the count of resistance (*r_i,j_*) among tested *Enterobacterales* isolates (*n_i,j_*) using a probability of resistance (*p_i,j_*), which is modelled as:

$$r_{i,j}\sim Binomial\left( p_{i,j},n_{i,j} \right)$$

$logit\left( p_{i,j} \right)=\alpha_{j}+\beta_{1,j}R_{CGC,i,j}+\beta_{2,j}R_{Tax,i}$(Equation 1)

The model intercept (𝛼_j_) is specific for each antibiotic (*j*), representing a baseline propensity of resistance for any given antibiotic. Because resistance propensities can vary widely between different antibiotics, we assumed independent baselines (fixed effects for 𝛼_j_, see priors below). The setting-specific information is *R_Tax_*_,_*_i_*, which gives information about pathogen levels in setting *i*, as well as *R_CGC_*_,_*_i_*_,_*_j_*, which carries information about resistance toward antibiotic *j* in setting *i*. Both *R_Tax_*_,_*_i_*  and *R_CGC_*_,_*_i_*_,_ are the z-transformed metrics as described above. For *β*_1_*_,_*_j_ and *β*_2_*_,_*_j_, the predictive effects of *R_CGC_* and *R_Tax_*, we assumed these to represent the clinical ecology of resistance genes so that they are specific to each antibiotic, *j*, but not to each setting, *i*. We further assumed that different antibiotics have different but related *β*-values (variable effects, specified below). We included only those antibiotics that had existing antibiotic susceptibility test (AST) data in at least two out of three settings (i.e. trimethoprim-sulfamethoxazole, nitrofurantoin, nalidixic acid, meropenem, imipenem, gentamicin, ciprofloxacin, chloramphenicol, cefuroxime, ceftriaxone, ceftazidime, cefpodoxime, cefoxitin, cefotaxime, ampicillin, amikacin); missing observations were excluded from the likelihood evaluation. Due to the limited number of infection isolates with AST results (especially in Cambodia), we chose standard weakly informative priors for the intercept (𝛼_j_) and the effect parameters (*β*_1_*_,_*_j_, *β*_2_*_,_*_j_). In addition, we restricted the effect of gene abundance to be positive, reflecting our view that only a positive association of resistance genes and clinical resistance is biologically reasonable. We therefore chose:

$$\beta_{1,j}\sim N^{+}(\mu_{\beta,1},\sigma_{\beta,1})$$

$$\beta_{2,j}\sim N(\mu_{\beta,2},\sigma_{\beta,2})$$

$$\alpha_{j}\sim N(0,1)$$

$$\mu_{\beta,1}\sim N^{+}\left( 0,1 \right)$$

$$\mu_{\beta,2}\sim N\left( 0,1 \right)$$

$$\sigma_{\beta,1}\sim N^{+}\left( 0,1 \right)$$

$$\sigma_{\beta,2}\sim N^{+}\left( 0,1 \right)$$

where *N* denotes a normal distribution and *N*^+^ denotes a half-normal distribution covering only positive values. Each model was fit using Stan software^22^ (v2.19.1), with which we sampled 50,000 samples after a burn-in period of 5,000 samples using four independent chains. We assessed chain convergence by inspecting chain traceplots and ensuring small values of the R-hat chain convergence diagnostic (R-hat<0.01)^23^.

The best taxonomy-adjusted AMR metric was selected using Bayesian leave-one-out cross validation^21^ as described in the main text. Three baseline models were considered in model comparisons as shown in the table S1 above.

## **Validation of pooling**

We evaluated to what extent resistome (i.e. the repertoire of AMR genes within a metagenome) data for pooled faecal extracts was a non-biased representation of the individual faecal resistomes making up the pool. Thirty high DNA-yield samples (≥300ng DNA/34ul) from each setting were used to create 30-sample pools for the validation study, as well as being sequenced individually and being included in the population pools. To prevent bias, systematic associations between high-yield samples and population traits were ruled out in advance. AMR gene abundances (CGCs) of the 30-sample pools and individually sequenced samples were converted to relative abundances, such that abundances across all AMR genes in each sample summed to one. Then, for each of the three different settings, individual samples were used to compute the empirical distribution of each AMR gene by repeated random sampling of its relative abundance out of the individual samples (bootstrapping with *n*=100,000 repeats). Because bootstrapping of gene abundances relies on having a sufficient number of samples with non-zero abundance, we limited our analysis to AMR genes present in ≥50% of all individual samples (n=121 genes). We then compared the pool abundance of each AMR gene with its empirical distribution in the same setting (within-setting comparison) and in the other two settings (across-setting comparison). We computed the fraction of AMR genes for which the pool abundance was within the 90% central quantile of the empirical distribution. The resulting value was restricted between 0 (i.e. 0% of AMR genes in the pool were as expected given the individual resistomes) and 1 (i.e. 100% of AMR genes in the pool were as expected). Given the central quantile choice above (i.e. 90%), a value of ~0.90 would imply a non-biased representation of individual resistomes by the pooled resistome.

For visualization, NMDS - an ordination-based method - was used to show pair-wise dissimilarities between resistomes from population pools, 30-sample-pools and individual samples within and across settings. NMDS was based on AMR profiling derived from rarefied sequencing data (see section 1.5 above).

# **Supplementary results**

## **Less common *Enterobacterales* in metagenomic population pools and in bloodstream and cerebrospinal fluid infections.**

Figure S1 shows relative abundances of less common *Enterobacterales* in metagenomic population pools and proportions of bloodstream and cerebrospinal fluid infections caused by less common *Enterobacterales* in Cambodian, Kenyan and UK study settings. Panels for metagenomic population pools (*1A*, *1B*) show, for each setting, the abundances of *Enterobacterales* taxa divided by the total abundance of bacterial taxa in a pool. Abundances are derived from Bracken estimates. Panels for invasive infection data (*2A*, *2B*) show the proportion of bloodstream and cerebrospinal fluid *Enterobacterales* isolates out of all bloodstream and cerebrospinal fluid isolates with speciation results in target age groups, in each setting, from 2010-2017 (Cambodia [n=197]; Kenya [n=910]; UK [n=3356]).

**Figure S1 Less common *Enterobacterales* in metagenomic population pools and in bloodstream and cerebrospinal fluid infections**

## **Main species and genera (all bacterial orders) identified from bloodstream and cerebrospinal fluid infections.**

Panels in figure S2 show, for each study setting, percentages of the most common bacterial species and genera out of all bacterial infection isolates with speciation results identified from blood and cerebrospinal fluid samples in target age groups, from 2010-2017 (Cambodia [n=197]; Kenya [n=910]; UK [n=3356]).

**Figure S2 Main species and genera (all bacterial orders) identified from bloodstream and cerebrospinal fluid infections.**

## **Bayesian model comparisons**

Table S2 shows for each of the nine models that are indicated in the far-left column, the out of sample prediction accuracy computed through Bayesian leave-one-out cross cross-validation^21^ (second column, loo_prediction, measured as expected log pointwise predictive density). The far-right column (loo_diff_to_bestmodel), gives for each model the difference in prediction accuracy compared to the best model, which is the one using *R_Tax_e4_* and *R_CGC_ALL_*. The 95% credible intervals of this differences are given in brackets and are computed using the standard error of the difference in prediction accuracy^21^.

**Table S2 Model comparisons**

| **Model** | **loo_prediction** | **loo_diff_to_bestmodel** |
| --- | --- | --- |
| *R_Tax_e4_* **&** *R_CGC_ALL_* ^1^ | -170.48 | 0 |
| *R_Tax_e4_* **&** *R_CGC_DEF_* | -174.31 | -3.84 [-18.39, 10.71] |
| *R_Tax_e_* **&** *R_CGC_ALL_* | -172.02 | -1.54 [-6.41, 3.33] |
| *R_Tax_e_* **&** *R_CGC_DEF_* | -178.59 | -8.12 [-22.17, 5.94] |
| *R_Tax_E_* **&** *R_CGC_ALL_* | -184.16 | -13.69 [-35.18, 7.81] |
| *R_Tax_E_* **&** *R_CGC_DEF_* | -189.98 | -19.5 [-42.28, 3.28] |
| Baseline: Null | -393.76 | -223.29 [-330, -116] |
| Baseline: *R_Tax_e4_* | -321.09 | -151 [-232, -69] |
| Baseline: *R_CGC_ALL_* | -356.76 | -186.28 [-281, -91] |

^1^ Best model.

## **Model predictions for the best model, compared to the null model**

Table S3 gives for each setting (UK, Cambodia, or Kenya) the number of culture isolates determined to be resistant to each antibiotic as well as the number of total cultures tested for this resistance (absence of testing routines is indicated with NA). The two columns on the right show the model predictions as mean count of predicted resistant cultures and 95% credible interval. Second from left is the best model (according to leave-one-out prediction accuracy) and next to it the null model (without metagenomic predictors). The best model accurately predicted the number of resistant infections for 100% of these antibiotics in Kenya (12/12) and UK (14/14) and for 75% of antibiotics (9/12) in Cambodia. The baseline model (i.e. no metagenomics information) correctly predicted resistance for 50% of antibiotics across the three settings (19/38).

**Table S3 Model predictions for the best model compared to the null model**

| **Setting** | **Antibiotic** | **Blood/Cerebrospinal fluid infection metadata** | | **Model predictions**  **(Mean [95% credible interval])** | |
| --- | --- | --- | --- | --- | --- |
|  |  | **Resistant culture isolates**  **(count)** | **Culture isolates with AST data**  **(count)** | ***R_Tax_e4_* & *R_CGC_ALL_*** | **Null model** |
| Cambodia | Amikacin | NA | NA | 82 (21 - 198) | 24 (14 - 37) |
| Cambodia | Ampicillin | 61 | 65 | 60 (55 - 65) | 45 (39 - 54) |
| Cambodia | Cefotaxime | NA | NA | 124 (100 - 150) | 83 (66 - 103) |
| Cambodia | Cefoxitin | NA | NA | 65 (48 - 87) | 42 (29 - 57) |
| Cambodia | Cefpodoxime | 35 | 62 | 23 (15 - 35) | 10 (5 - 16) |
| Cambodia | Ceftazidime | 23 | 53 | 15 (9 - 22) | 13 (8 - 20) |
| Cambodia | Ceftriaxone | 34 | 55 | 31 (23 - 42) | 14 (8 - 21) |
| Cambodia | Cefuroxime | NA | NA | 120 (89 - 154) | 146 (123 - 171) |
| Cambodia | Chloramphenicol | 23 | 58 | 26 (18 - 36) | 15 (9 - 22) |
| Cambodia | Ciprofloxacin | 24 | 63 | 12 (7 - 19) | 13 (8 - 21) |
| Cambodia | Gentamicin | 29 | 62 | 26 (17 - 38) | 11 (7 - 19) |
| Cambodia | Imipenem | 1 | 58 | 2 (0 - 6) | 0 (0 - 3) |
| Cambodia | Meropenem | 0 | 3 | 0 (0 - 1) | 0 (0 - 0) |
| Cambodia | Nalidixic Acid | 2 | 2 | 1 (0 - 2) | 1 (0 - 2) |
| Cambodia | Nitrofurantoin | 9 | 45 | 10 (5 - 18) | 11 (5 - 18) |
| Cambodia | Trim-Sulfa^1^ | 43 | 64 | 24 (17 - 32) | 28 (21 - 37) |
| Kenya | Amikacin | 3 | 304 | 4 (0 - 10) | 12 (5 - 19) |
| Kenya | Ampicillin | 212 | 324 | 212 (189 - 236) | 226 (207 - 250) |
| Kenya | Cefotaxime | 63 | 324 | 63 (46 - 85) | 98 (80 - 120) |
| Kenya | Cefoxitin | 22 | 306 | 22 (11 - 35) | 48 (34 - 64) |
| Kenya | Cefpodoxime | NA | NA | 415 (406 - 419) | 72 (55 - 91) |
| Kenya | Ceftazidime | 62 | 308 | 63 (46 - 85) | 79 (65 - 99) |
| Kenya | Ceftriaxone | 60 | 308 | 60 (43 - 81) | 79 (61 - 101) |
| Kenya | Cefuroxime | 1 | 15 | 1 (0 - 4) | 4 (2 - 8) |
| Kenya | Chloramphenicol | 67 | 323 | 67 (49 - 88) | 84 (66 - 106) |
| Kenya | Ciprofloxacin | 68 | 324 | 70 (51 - 91) | 70 (55 - 87) |
| Kenya | Gentamicin | 72 | 324 | 72 (53 - 93) | 61 (47 - 77) |
| Kenya | Imipenem | 1 | 311 | 1 (0 - 6) | 5 (1 - 12) |
| Kenya | Meropenem | NA | NA | 180 (0 - 444) | 5 (1 - 11) |
| Kenya | Nalidixic Acid | NA | NA | 18 (3 - 26) | 18 (13 - 23) |
| Kenya | Nitrofurantoin | NA | NA | 9 (0 - 31) | 10 (5 - 18) |
| Kenya | Trim-Sulfa^1^ | 200 | 324 | 202 (181 - 228) | 142 (124 - 164) |
| UK | Amikacin | 42 | 903 | 43 (27 - 61) | 35 (22 - 52) |
| UK | Ampicillin | NA | NA | 185 (176 - 194) | 135 (122 - 152) |
| UK | Cefotaxime | 103 | 224 | 102 (84 - 124) | 68 (53 - 84) |
| UK | Cefoxitin | 61 | 224 | 60 (44 - 81) | 35 (24 - 49) |
| UK | Cefpodoxime | 108 | 776 | 117 (90 - 145) | 133 (106 - 162) |
| UK | Ceftazidime | 243 | 906 | 250 (215 - 288) | 235 (205 - 271) |
| UK | Ceftriaxone | 0 | 3 | 1 (0 - 3) | 0 (0 - 2) |
| UK | Cefuroxime | 291 | 908 | 290 (254 - 331) | 288 (252 - 329) |
| UK | Chloramphenicol | 14 | 20 | 11 (7 - 16) | 5 (2 - 9) |
| UK | Ciprofloxacin | 189 | 912 | 199 (166 - 234) | 198 (168 - 231) |
| UK | Gentamicin | 144 | 905 | 146 (119 - 179) | 172 (143 - 203) |
| UK | Imipenem | NA | NA | 11 (0 - 27) | 3 (0 - 7) |
| UK | Meropenem | 7 | 908 | 8 (2 - 17) | 11 (4 - 21) |
| UK | Nalidixic Acid | 35 | 49 | 35 (28 - 44) | 34 (27 - 43) |
| UK | Nitrofurantoin | 11 | 41 | 9 (4 - 17) | 10 (5 - 17) |
| UK | Trim-Sulfa^1^ | 168 | 549 | 184 (156 - 214) | 241 (214 - 271) |

^1^ “Trim-Sulfa” is short for trimethoprim-sulfamethoxazole.

## **Bayesian model prediction of proportion of *Enterobacterales* invasive infections with resistance to antibiotics**

Predictions are shown for antibiotics where antibiotic susceptibility test (AST) results were available from > 100 invasive infection isolates (Available only from UK and Kenya, for a total of 14 antibiotics). Horizontal bars represent 95% highest density posterior interval and vertical lines represent means of the model predictions based on metagenomic data from population pools. Red circles show the proportion of blood and cerebrospinal fluid *Enterobacterales* infections that were found to be resistant to the antibiotic listed in the y-axis. The number of isolates with AST results are also given in the y-axis. Percentages were calculated by dividing observed and predicted counts by the total number of invasive infection isolates with AST data in each setting (see y-axis). “Trim-Sulfa” is short for trimethoprim-sulfamethoxazole; “Cloramph” is short for chloramphenicol. Predictions were accurate for 100% of antibiotics (14/14).

**Figure S3 Bayesian model prediction of proportion of *Enterobacterales* invasive infections with resistance to antibiotics**

## **Pooling faecal DNA extracts as a strategy to measure population-level AMR gene distributions**

Pair-wise dissimilarities (Bray-Curtis) in resistomes (CGC) from population pools, 30-sample-pools and individual samples were calculated both within and across settings. Population pools, 30-sample pools and individual samples were less dissimilar and hence more closely related within settings than across settings (figure S4). Within each setting, 30-sample-pool resistomes had substantially higher similarity to individual resistomes from the same setting relative to the comparison with other settings (mean Bray-Curtis dissimilarities between 30-sample-pools and individuals, individuals and population-pools, 30-sample-pools and population-pools were 0.029, 0.153, 0.152 respectively). In Cambodia, 362, 482 and 375 AMR genes were identified in the 30-sample pool, population-pool and 30 individual samples respectively. In Kenya, 337, 382 and 340 AMR genes were identified in the 30-sample pool, population-pool and 30 individual samples respectively. Finally, in UK, 317, 386 and 319 AMR genes were identified in the 30-sample pool, population-pool and 30 individual samples respectively.

When comparing individual samples and pools from the same setting quantitatively (validation of pooling; for methods see section 1.7 above), the average fraction of AMR genes for which the 30-sample pool estimate was within the central interval of the empirical distribution inferred from individually sequenced samples was 97% (Kenya: 98%; Cambodia: 97%; UK: 95%). In contrast, the average fraction was 86% across comparisons between different settings (min-max: 80-92%). All 30-sample pool resistomes therefore had substantially higher similarity to individual resistomes from the same setting relative to the comparison with other settings, consistent with results from the NMDS analysis.

**Figure S4 Non-metric multidimensional scaling (NMDS) plot and dendrogram showing pair-wise dissimilarities (Bray-Curtis) of resistance gene counts from population pools (PP), 30-sample-pools (30S) and individual samples (SI) within and between settings.**


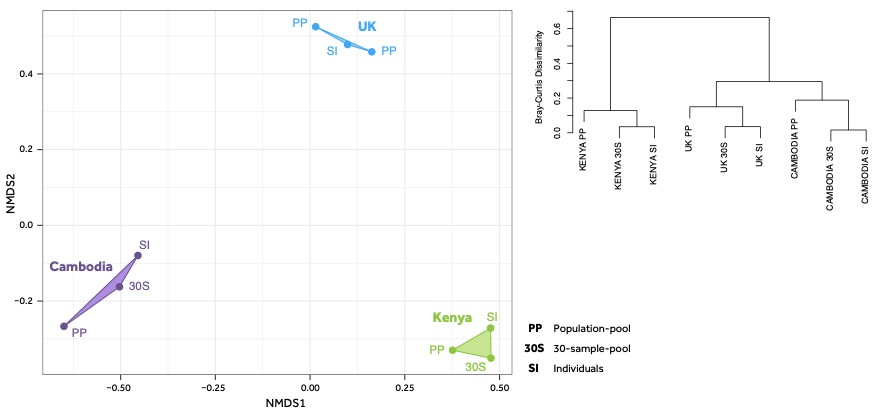


**Table S4 Pair-wise dissimilarities (Bray-Curtis) between all samples.**

|  | Cambodia-30 | Cambodia-Pop | Cambodia-Ind | Kenya-30 | Kenya-Pop | Kenya-Ind | UK-30 | UK-Pop |
| --- | --- | --- | --- | --- | --- | --- | --- | --- |
| Cambodia-Pop | 0.185 |  |  |  |  |  |  |  |
| Cambodia-Ind | 0.016 | 0.188 |  |  |  |  |  |  |
| Kenya-30 | 0.638 | 0.567 | 0.638 |  |  |  |  |  |
| Kenya-Pop | 0.664 | 0.593 | 0.664 | 0.128 |  |  |  |  |
| Kenya-Ind | 0.656 | 0.588 | 0.656 | 0.035 | 0.121 |  |  |  |
| UK-30 | 0.292 | 0.232 | 0.292 | 0.487 | 0.512 | 0.508 |  |  |
| UK-Pop | 0.189 | 0.223 | 0.187 | 0.552 | 0.585 | 0.572 | 0.143 |  |
| UK-Ind | 0.294 | 0.227 | 0.295 | 0.479 | 0.502 | 0.500 | 0.035 | 0.150 |

# **References**

1 Standing JF, Ongas MO, Ogwang C, *et al.* Dosing of Ceftriaxone and Metronidazole for Children With Severe Acute Malnutrition. *Clin Pharmacol Ther* 2018; **104**: 1165–74.

2 Turner P, Pol S, Soeng S, *et al.* High prevalence of antimicrobial-resistant gram-negative colonization in hospitalized cambodian infants. *Pediatr Infect Dis J* 2016; **35**: 856–61.

3 Otter JA, Dyakova E, Bisnauthsing KN, *et al.* Universal hospital admission screening for carbapenemase-producing organisms in a low-prevalence setting. *J Antimicrob Chemother* 2016; **71**. http://www.jac.oxfordjournals.org/lookup/doi/10.1093/jac/dkw309 (accessed Aug 22, 2016).

4 Dyakova E, Bisnauthsing KN, Querol-Rubiera A, *et al.* Efficacy and acceptability of rectal and perineal sampling for identifying gastrointestinal colonisation with ESBL-Enterobacteriaceae. 2017 DOI:10.1016/j.cmi.2017.02.019.

5 Otter JA, Natale A, Batra R, *et al.* Individual- and community-level risk factors for ESBL Enterobacteriaceae colonization identified by universal admission screening in London. *Clin Microbiol Infect* 2019; **0**. DOI:10.1016/j.cmi.2019.02.026.

6 Chheng K, Carter MJ, Emary K, *et al.* A Prospective Study of the Causes of Febrile Illness Requiring Hospitalization in Children in Cambodia. *PLoS One* 2013; **8**. DOI:10.1371/journal.pone.0060634.

7 Fox-Lewis A, Takata J, Miliya T, *et al.* Antimicrobial resistance in invasive bacterial infections in hospitalized children, Cambodia, 2007–2016. *Emerg Infect Dis* 2018; **24**: 841–51.

8 Scott JAG, Bauni E, Moisi JC, *et al.* Profile: The Kilifi health and demographic surveillance system (KHDSS). *Int J Epidemiol* 2012; **41**: 650–7.

9 Tosas Auguet O, Stabler RA, Betley J, *et al.* Frequent Undetected Ward-Based Methicillin-Resistant Staphylococcus aureus Transmission Linked to Patient Sharing Between Hospitals. *Clin Infect Dis* 2018; **66**: 840–8.

10 British Society for Antimicrobial Chemotherapy. http://www.bsac.org.uk/ (accessed Oct 29, 2019).

11 Clinical and Laboratory Standards Institute (CLSI). Performance Standards for Antimicrobial Susceptibility Testing. CLSI document M100-S27., 27th edn. Wayne, PA: Clinical and Laboratory Standards Institute, 2017.

12 Satinsky BM, Gifford SM, Crump BC, Moran MA. Chapter Twelve – Use of Internal Standards for Quantitative Metatranscriptome and Metagenome Analysis. In: Methods in Enzymology. 2013: 237–50.

13 Lamble S, Batty E, Attar M, *et al.* Improved workflows for high throughput library preparation using the transposome-based nextera system. *BMC Biotechnol* 2013; **13**. DOI:10.1186/1472-6750-13-104.

14 Gweon HS, Shaw LP, Swann J, *et al.* The impact of sequencing depth on the inferred taxonomic composition and AMR gene content of metagenomic samples. *Environ Microbiome* 2019; **14**: 7.

15 Babraham Bioinformatics - Trim Galore! http://www.bioinformatics.babraham.ac.uk/projects/trim_galore/ (accessed Oct 28, 2019).

16 Wood DE, Salzberg SL, Venter C, *et al.* Kraken: ultrafast metagenomic sequence classification using exact alignments. *Genome Biol* 2014; **15**: R46.

17 Lu J, Breitwieser FP, Thielen P, Salzberg SL. Bracken: Estimating species abundance in metagenomics data. *PeerJ* 2017; **2017**. DOI:10.7717/peerj-cs.104.

18 Jia B, Raphenya AR, Alcock B, *et al.* CARD 2017: Expansion and model-centric curation of the comprehensive antibiotic resistance database. *Nucleic Acids Res* 2017; **45**: D566–73.

19 Alcock BP, Raphenya AR, Lau TTY, *et al.* CARD 2020: antibiotic resistome surveillance with the comprehensive antibiotic resistance database. *Nucleic Acids Res* 2019; published online Oct 29. DOI:10.1093/nar/gkz935.

20 BBMap: A Fast, Accurate, Splice-Aware Aligner (Conference) | OSTI.GOV. https://www.osti.gov/biblio/1241166 (accessed Oct 28, 2019).

21 Vehtari A, Gelman A, Gabry J. Practical Bayesian model evaluation using leave-one-out cross-validation and WAIC. *Stat Comput* 2017; **27**: 1413–32.

22 Carpenter B, Gelman A, Hoffman MD, *et al.* Stan: A probabilistic programming language. *J Stat Softw* 2017; **76**. DOI:10.18637/jss.v076.i01.

23 Gelman A, Rubin DB. Inference from Iterative Simulation Using Multiple Sequences. Stat. Sci. ; **7**: 457–72.

1. NB - this step is not absolutely essential. This is used in our metagenomics workflows for individual samples, where it is of particular value in the normalisation of gene counts (please see Gweon et al, Environmental Microbiome volume 14, Article number: 7 (2019), https://environmentalmicrobiome.biomedcentral.com/articles/10.1186/s40793-019-0347-1). [↑](#footnote-ref-1)
2. Rectal Swabs in PBS were processed at the Nuffield Department of Medicine (University of Oxford, UK) using the FastPrep-24 5G instrument (MP Biomedicals, Santa Ana, CA, USA). Rectal swabs in ATM were processed at the Clinical Infection and Diagnostics Research laboratory at Guy’s and St Thomas’ NHS Foundation Trust, UK. A FastPrep instrument or equivalent was not available in the latter. Bead beating steps were hence replaced by vortexing at maximum speed on the Labnet VX-100 vortex for 30 minutes. [↑](#footnote-ref-2)
3. NB - this step is not absolutely essential. This is used in our metagenomics workflows for individual samples, where it is of particular value in the normalisation of gene counts (please see Gweon et al, Environmental Microbiome volume 14, Article number: 7 (2019), https://environmentalmicrobiome.biomedcentral.com/articles/10.1186/s40793-019-0347-1). [↑](#footnote-ref-3)
